# Characterization of histological changes at the tillering stage (Z21) in resistant and susceptible wheat plants infected by *Tilletia controversa* Kühn

**DOI:** 10.1186/s12870-020-02819-0

**Published:** 2021-01-18

**Authors:** Tongshuo Xu, Dandan Qin, Ghulam Muhae Ud Din, Taiguo Liu, Wanquan Chen, Li Gao

**Affiliations:** State Key Laboratory for Biology of Plant Disease and Insect Pests, Institute of Plant Protection, Chinese Academy of Agricultural Sciences, Beijing, 100193 China

**Keywords:** *Tilletia controversa* Kühn, Wheat dwarf bunt, Transmission electron microscopy, Scanning electron microscopy, Histology

## Abstract

**Background:**

Dwarf bunt, which is caused by *Tilletia controversa* Kühn, is a soilborne and seedborne disease that occurs worldwide and can lead to 70% or even total losses of wheat crops. However, very little information is available about the histological changes that occur in dwarf bunt-resistant and dwarf bunt-susceptible wheat plants at the tillering stage (Z21). In this study, we used scanning electron microscopy and transmission electron microscopy to characterize the histological changes at this stage in resistant and susceptible wheat cultivars infected by *T. controversa*.

**Results:**

Using scanning electron microscopy, the root, stem, and leaf structures of resistant and susceptible cultivars were examined after *T. controversa* infection. The root epidermal and vascular bundles were more severely damaged in the susceptible *T. controversa*-infected plants than in the resistant plants. The stem cell and longitudinal sections were much more extensively affected in susceptible plants than in resistant plants after pathogen infection. However, slightly deformed mesophyll cells were observed in the leaves of susceptible plants. With transmission electron microscopy, we found that the cortical bundle cells and the cell contents and nuclei in the roots were more severely affected in the susceptible plants than in the resistant plants; in the stems and leaves, the nuclei, chloroplasts, and mesophyll cells changed significantly in the susceptible plants after fungal infection. Moreover, we found that infected susceptible and resistant plants were affected much more severely at the tillering stage (Z21) than at the seedling growth stage (Z13).

**Conclusion:**

Histological changes in the wheat roots, stems and leaves were much more severe in *T. controversa*-infected susceptible plants than in infected resistant plants at the tillering stage (Z21).

**Supplementary Information:**

The online version contains supplementary material available at 10.1186/s12870-020-02819-0.

## Background

Wheat is a critical food crop that plays a pivotal role in worldwide food security [1]. Dwarf bunt of wheat is one of the most serious diseases of this crop in the world, and it is a quarantine disease in many countries [2–4]. *T. controversa*, a soilborne and seedborne fungal pathogen, is the causal agent of dwarf bunt of wheat. *T. controversa* has a wide host range but primarily damages the genus *Triticum*; *Hordeum vulgare* and rye are also affected. To date, more than 70 species of plants in 18 genera within the Gramineae family are known to be affected [5, 6]. Usually, losses due to dwarf bunt reach 10–20%, but under severe conditions, losses can reach 70–80% or even complete crop failure [7].

The plant vascular system performs two vital functions, namely the delivery of resources (essential mineral nutrients, water, amino acids and sugars) to the various plant organs and the provision of mechanical support [8]. In addition, the vascular bundle serves as an effective long-distance communication system, with the xylem and phloem bringing in information relating to biotic and abiotic conditions below and above the ground, respectively [9]. Mesophyll cells contain a large population of chloroplast organelles and therefore are very important for photosynthesis in higher plants [10]. The nucleus contains most of the genetic material of the cell [11], and chloroplasts play an important role in photosynthesis [12]; both are very important organelles in plants. The infection of these organelles by fungal pathogens may lead to damage to their structure and function [13]. Previous studies showed that *Verticillium dahliae* conidia could not penetrate the cuticles of resistant lettuce cultivars, but the plasma membrane and cytoplasm were severely infected in a susceptible cultivar [14]. An occluding material was produced in inoculated resistant cultivars (Manteigão Fosco 11 and VP8) while not found in inoculated susceptible cultivar (Meia Noite) and non-inoculated control of both resistant and susceptible cultivars, which may help defend against infection by *Fusarium oxysporum Schlecht*. f. sp. *phaseoli* in the resistant cultivars [15]. In barley, *Rhynchosporium secalis* (a smut pathogen) infects the plasma membrane and the cytoplasmic materials of susceptible cultivars, but a dense osmiophilic layer develops inside the wall of resistant cultivars [16]. Similarly, *Sphacelotheca reiliana* only infects maize seedlings in susceptible cultivars and creates necrotic symptoms [17]. Colonization is limited to the lower stem portion in the resistant cultivar, whereas the xylem of the susceptible cultivar is quickly and intensively colonized in other crops [18, 19]. In the resistant cultivar parenchyma, the cells surrounding the colonized vascular tissues show rapid cytoplasmic disturbance and increased metabolic activity [20].

Hansen et al. [21] studied histopathology in inoculated wheat plants of both susceptible and resistant cultivars, they focused on the development of the fungal hyphae from inoculation of the host plant up to spore formation and found that the spread of the pathogens leading to dwarf bunt, rye smut and common smut in resistant plants was usually retarded compared to the spread and development of the pathogen in the tissues of susceptible plants. They also reported that the mycelia of dwarf bunt were able to penetrate wounded seedlings of resistant host varieties and species but fail to spread further in the tissues. Similarly, Woolman [22] demonstrated that the process of the infection caused by *Tilletia tritici* (leading to wheat common bunt) in resistant cultivars was usually slow compared to the aggressive spread in susceptible cultivars, and they were mainly interested in the three stages of infection: the entrance of the hyphae into the epidermal cell and its development, the development in the deeper parts of the coleoptile and in the sheath tissues of the earliest true leaves, and the development in the very young leaf blades and in the nodes, internodes, and growing points of the plant. Fernandez et al. [23] reported on the differences of intercellular hyphae that were reamplified throughout the primordial leaf and nodal tissue and reached the cells of the growing point between cultivars susceptible and resistant to *T. controversa* (the pathogen causing dwarf bunt), and they found that the development and spread of the hyphae is usually slow in resistant cultivars. Those studies mentioned above used only light microscopy or sectioning for anatomical investigations. However, in this study, with scanning electron microscopy and transmission electron microscopy, we identified the more important growth stages by analyzing the tillering (Z21) and seedling growth (Z13) [24] stages for histological changes in the root, stem and leaf cells in response to *T. controversa* infection in resistant and susceptible wheat cultivars.

## Results

### Detection of *T. controversa* in plants

The stages of seedling growth (Z13) and tillering (Z21) in both infected and control plants of resistant and susceptible cultivars were shown in Additional file 1. We detected the pathogen of *T. controversa* in the plants by combining microscopy and molecular detection methods. For the detection of *T. controversa* by confocal laser scanning microscopy, both in the stages of seedling growth (Z13) and tillering (Z21), we found the hyphae of *T. controversa* in both the infected resistant and susceptible wheat cultivars in the roots, stems, and leaves (Additional file 2). For molecular detection, we extracted DNA from leaves of the inoculated and control plants of both cultivars at both stages to detect the pathogen. The expected 372 bp fragment was detected in the infected leaf samples (Additional file 3).

### Comparison of root, stem, and leaf tissue structures of resistant and susceptible plants with scanning electron microscopy

**Fig. 1:**
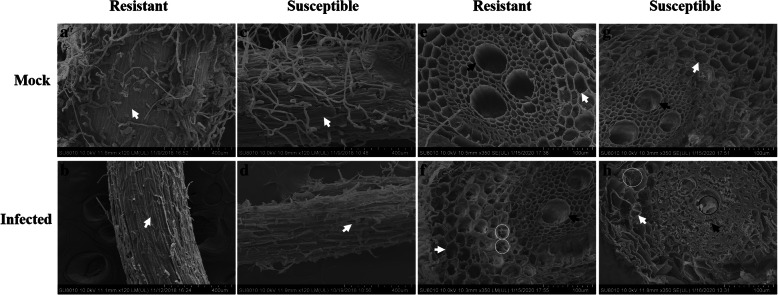
Histological characteristics of the roots of the mock and infected resistant and susceptible cultivars at the tillering stage (Z21) under scanning electron microscopy. **a** Epidermal cells of the mock resistant cultivar. **b** Epidermal cells of the infected resistant cultivar. **c** Epidermal cells of the mock susceptible cultivar. **d** Epidermal cells of the infected susceptible cultivar. **e** Vascular bundle cells and cortical parenchyma cells of the mock resistant cultivar. **f** Vascular bundle cells and cortical parenchyma cells of the infected resistant cultivar. **g** Vascular bundle cells and cortical parenchyma cells of the mock susceptible cultivar. **h** Vascular bundle cells and cortical parenchyma cells of the infected susceptible cultivar. The resistant cultivar was Mianyang 26/Yumai 47, and the susceptible cultivar was CU42. White arrows in (**a**-**d**) indicate epidermal cells, white arrows in (**e**-**h**) indicate cortical parenchyma cells, black arrows indicate vascular bundle cells, white circles in (**f** and **h**) indicate hyphae in cortical parenchyma cells, and black circles in (**h**) indicates hyphae in vascular bundle cells

Based on the resistant (Mianyang 26/Yumai 47) and susceptible cultivar (CU42), in the root cells, as shown in Fig. 1, there were few differences between the epidermal cells of the resistant cultivar and those in the mock treatment (Fig. 1a, b); however, in the inoculated susceptible cultivar, the epidermal cells were severely infected, and some damage appeared on the epidermal cells (Fig. 1d). In the mock treatment, the epidermal cells were closely packed (Fig. 1c). We found fungal hyphae in the cortical parenchyma cells of the infected resistant cultivar (Fig. 1f) but not in the mock resistant cultivar (Fig. 1e). Moreover, we found hyphae in both the vascular cells and cortical parenchyma cells of the infected susceptible cultivar (Fig. 1h) but not in those of the mock susceptible cultivar (Fig. 1g).

**Fig. 2:**
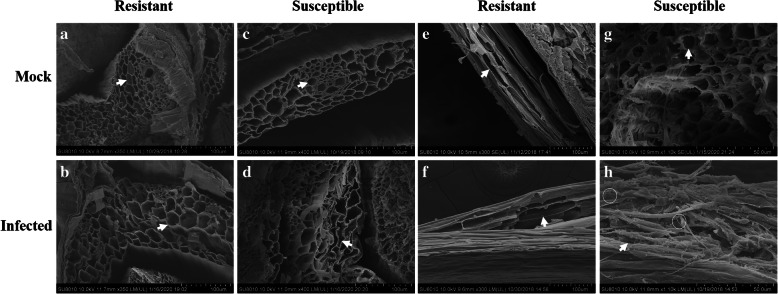
Histological characteristics of the stems of the mock and infected resistant and susceptible cultivars at the tillering stage (Z21) under scanning electron microscopy. The first internodes (just above the roots) of the plants were examined. **a** Stem cell structure of the mock resistant cultivar. **b** Stem cell structure of the infected resistant cultivar. **c** Stem cell structure of the mock susceptible cultivar. **d** Stem cell structure of the infected susceptible cultivar. **e** Longitudinal section of the stem of the mock resistant cultivar. **f** Longitudinal section of the stem of the infected resistant cultivar. **g** Longitudinal section of the stem of the mock susceptible cultivar. **h** Longitudinal section of the stem of the infected susceptible cultivar. The resistant cultivar was Mianyang 26/Yumai 47, and the susceptible cultivar was CU42. White arrows in (**a**-**d**) indicate stem cells, white arrows in (**e**-**h**) indicate longitudinal section stem cells, and white circles in (**h**) indicate hyphae in the cell

For the stem cells, the first internodes (just above the roots) of the plants were examined. As shown in Fig. 2, there were no obvious differences in the stem cells between the infected resistant cultivar and the mock treatments (Fig. 2a, b). By contrast, the stem vascular system of the inoculated susceptible cultivar was severely affected, and most stem cells changed their shape compared to those in the mock susceptible cultivar (Fig. 2c, d). We also investigated longitudinal sections of both cultivars for more detail. In the resistant cultivar, whether mock (Fig. 2e) or infected (Fig. 2f), the cell structures were not destroyed by the pathogen. However, the fungus successfully colonized, ruptured and deformed the stem cells in the inoculated susceptible cultivar (Fig. 2h) but not those in the mock susceptible cultivar (Fig. 2g).

**Fig. 3:**
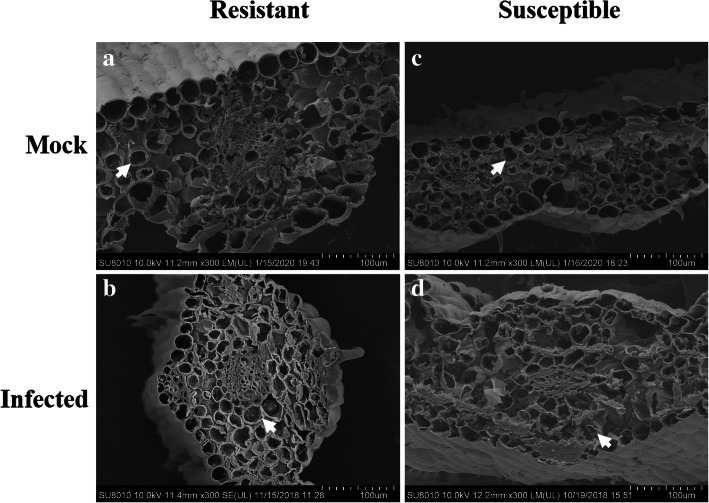
Histological characteristics of the leaves of the mock and infected resistant and susceptible cultivars at the tillering stage (Z21) under scanning electron microscopy. **a** Mesophyll cells of the mock resistant cultivar. **b** Mesophyll cells of the infected resistant cultivar. **c** Mesophyll cells of the mock susceptible cultivar. **d** Mesophyll cells of the infected susceptible cultivar. The resistant cultivar was Mianyang 26/Yumai 47, and the susceptible cultivar was CU42. White arrows in (**a**-**d**) indicate mesophyll cells

In Fig. 3, the results showed that the resistant cultivar tolerated the pathogenic effects of *T. controversa,* and few differences were observed in the mesophyll cells of either the infected resistant cultivar or the infected susceptible cultivar compared to their respective controls. For the histological characteristics of the leaves of infected resistant and susceptible cultivars at the tillering stage (Z21) (Fig. 3b, d), in comparison to those receiving mock treatments (Fig. 3a, c), few differences, including slight deformations of the mesophyll cells, were found between the infected susceptible cultivar (Fig. 3d) and the infected resistant cultivar (Fig. 3b). All the above results indicated that *T. controversa* only slightly infected the susceptible cultivar. Additionally, the morphology of the resistant and susceptible cultivar roots, stems and leaves was examined at the seedling growth stage (Z13). However, the growth and development of hyphae in these tissues were very slow (Additional files 4, 5 and 6). Specifically, we found that the root epidermal cells in infected resistant and susceptible cultivars did not show obvious changes compared to those in the mock treatments (Additional file 4a, b, c, d), while the cell walls of the vascular bundle cells of the infected susceptible cultivar were uneven compared to those in the mock treatment, and we also compared the effects on the infected resistant cultivar and the mock resistant cultivar (Additional file 4e, f, g, h). Some deformities were observed in the stem cells of the infected susceptible cultivar but not in the mock susceptible cultivar (Additional file 5), and slight damage occurred in the mesophyll cells of the infected susceptible cultivar, but not in the mock susceptible cultivar (Additional file 6). Therefore, the results indicated that the pathogen had much more extensive effects on the susceptible cultivar at the tillering stage (Z21) than at the seedling growth stage (Z13).

Another resistant (Yinong 18/Lankao 8) and susceptible cultivars (Dongxuan 3) were also observed. At the tillering stage (Z21), the root morphology of the resistant variety showed few differences between the mock and *T. controversa*-infected treatments (Additional file 7a, b, e, f). For the susceptible cultivar, the root cells showed damage in the infected plants (Additional file 7c, d, g, h). The vascular bundle cells, parenchyma cells (Additional file 7d), and root epidermal cells (Additional file 7h) were damaged, the root hairs were sparse and hyphae were found in the parenchyma cells (Additional file 7d). For the stem cells, the morphology of the resistant cultivar did not display any differences between the mock and infected treatments (Additional file 8a, b, e, f), while for the susceptible cultivar, which showed severely deformed cells in the infected compared to the mock treatment (Additional file 8c, d, g, h). The mesophyll cells of the susceptible and resistant cultivars displayed few differences (Additional file 9). In addition, at the seedling growth stage (Z13) in the root cells, both the resistant and susceptible cultivars showed few differences between the mock and *T. controversa*-infected treatments (Additional file 10a-h). For the stem cells, the infected susceptible cultivar showed cell abnormalities (Additional file 11c, d) that were not found in the resistant cultivar (Additional file 11a, b). The mesophyll cells of the susceptible varieties after infection were abnormal (Additional file 12c, d), unlike the resistant varieties, which did not show any differences after infection (Additional file 12a, b). Therefore, the results showed that *T. controversa* had more effects on the susceptible cultivar at the tillering stage (Z21) than at the seedling growth stage (Z13).

### Comparison of root, stem, and leaf tissue structures of resistant and susceptible plants under transmission electron microscopy

**Fig. 4:**
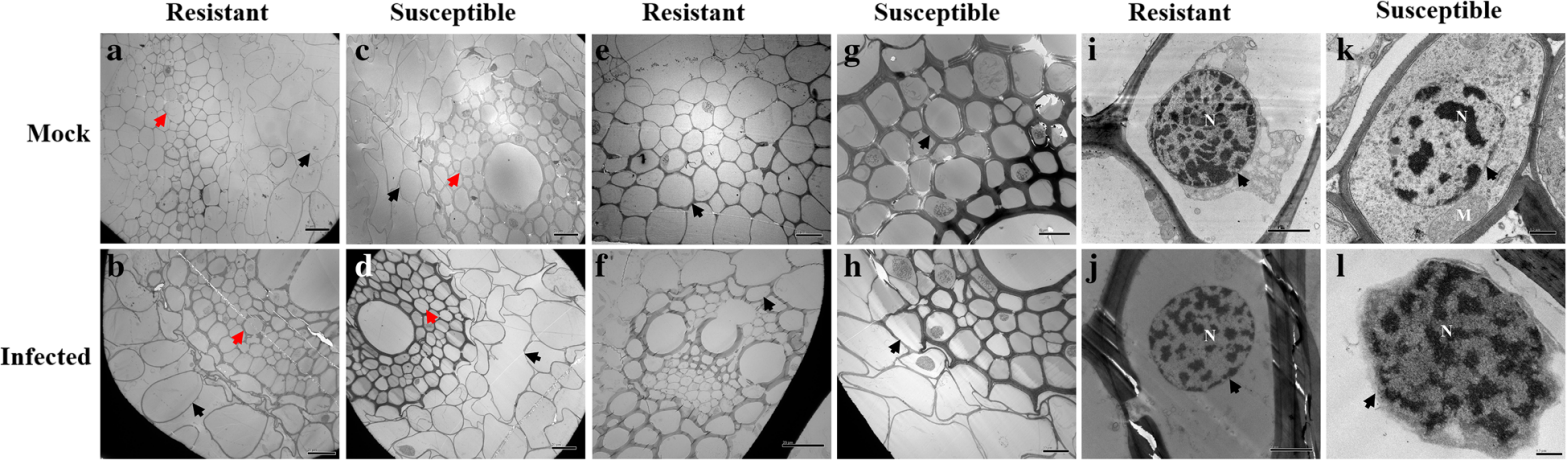
Histological characteristics of the roots of the mock and infected resistant and susceptible cultivars at the tillering stage (Z21) under transmission electron microscopy. **a** Vascular bundle cells and cortical parenchyma cells of the mock resistant cultivar. **b** Vascular bundle cells and cortical parenchyma cells of the infected resistant cultivar. **c** Vascular bundle cells and cortical parenchyma cells of the mock susceptible cultivar. **d** Vascular bundle cells and cortical parenchyma cells of the infected susceptible cultivar. **e** Root cell contents of the mock resistant cultivar. **f** Root cell contents of the infected resistant cultivar. **g** Root cell contents of the mock susceptible cultivar. **h** Root cell contents of the infected susceptible cultivar. **i** Root nucleus of the mock resistant cultivar. **j** Root nucleus of the infected resistant cultivar. **k** Root nucleus of the mock susceptible cultivar. **l** Root nucleus of the infected susceptible cultivar. The resistant cultivar was Mianyang 26/Yumai 47 and the susceptible cultivar was CU42. Black arrows in (**a**-**d**) indicate cortical parenchyma cells, red arrows in (**a**-**d**) indicate vascular bundle cells, black arrows in (**e**-**h**) indicate root cell contents, and black arrows in (**i**-**l**) indicate root nuclei (N: Nucleus; M: Mitochondrion; scale bar of (**a**-**f**) = 20 μm; scale bar of (**g** and **h**) =10 μm; scale bar of (**i** and **j**) =2 μm; scale bar of (**k** and **l**) =0.5 μm)

For the resistant (Mianyang 26/Yumai 47) and susceptible cultivar (CU42), in the root cells (Fig. 4), the cortical parenchyma cells were much more severely deformed than the vascular bundle cells in infected plants than in the corresponding mock plants, regardless of the cultivar (Fig. 4a, b, c, d). The pathogen intensively damaged the root cell contents and cell walls of the diminished tissues in the infected susceptible cultivar but not in the mock susceptible cultivar (Fig. 4g, h); this phenomenon was not found in infected resistant plants or in the mock resistant plants (Fig. 4e, f). Moreover, the root nucleus was degraded and the nuclear envelopes were ruptured in the inoculated susceptible cultivar but not in the mock susceptible cultivar; this phenomenon was not observed in the infected resistant plants or in the mock resistant plants (Fig. 4i, j, k, l).

**Fig. 5:**
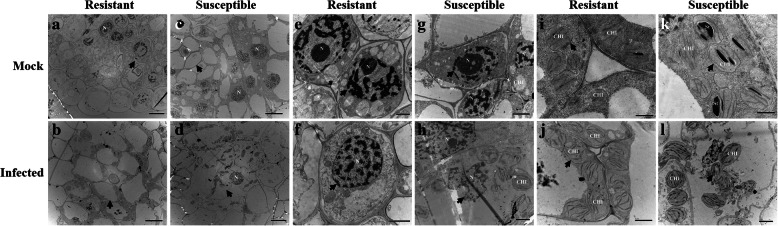
Histological characteristics of the stems of the mock and infected resistant and susceptible cultivars at the tillering stage (Z21) under transmission electron microscopy. **a** Stem cell structure of the mock resistant cultivar. **b** Stem cell structure of the infected resistant cultivar. **c** Stem cell structure of the mock susceptible cultivar. **d** Stem cell structure of the infected susceptible cultivar. **e** Stem cell nucleus of the mock resistant cultivar. **f** Stem cell nucleus of the infected resistant cultivar. **g** Stem cell nucleus of the mock susceptible cultivar. **h** Stem cell nucleus of the infected susceptible cultivar. **i** Stem chloroplast of the mock resistant cultivar. **j** Stem chloroplast of the infected resistant cultivar. **k** Stem chloroplast of the mock susceptible cultivar. **l** Stem chloroplast of the infected susceptible cultivar. The resistant cultivar was Mianyang 26/Yumai 47, and the susceptible cultivar was CU42. Black arrows in (**a**-**d**) indicate stem cells, black arrows in (**e**-**h**) indicate stem nuclei, and black arrows in (**i**-**l**) indicate stem chloroplasts (N: Nuclei; CHl: Chloroplast; St: Starch granule; scale bar of (**a**-**d**) =10 μm; scale bar of (**e**-**l**) = 2 μm)

In the stem cells (Fig. 5), the tissue morphology did not show major differences between the inoculated resistant plants and the mock plants (Fig. 5a, b). However, in the inoculated susceptible plants, the cells were deformed, scattered, and ruptured (Fig. 5d) compared to those in the mock plants (Fig. 5c). Similarly, the stem nuclei in the resistant cultivar showed few changes compared to those in the mock treatment (Fig. 5e, f). The nuclei in the inoculated susceptible cultivar were deformed, and the nuclear envelopes were ruptured and abnormal (Fig. 5h) compared to those in the mock treatment (Fig. 5g). Additionally, there were no obvious differences in the chloroplasts between the resistant and mock plants (Fig. 5i, j). However, degraded, and ruptured chloroplasts were observed in the inoculated susceptible cultivar but not in the mock treatment (Fig. 5k, l).

**Fig. 6:**
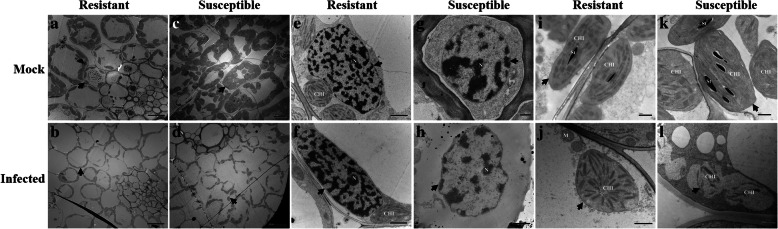
Histological characteristics of the leaves of the mock and infected resistant and susceptible cultivars at the tillering stage (Z21) under transmission electron microscopy. **a** Mesophyll cell structure of the mock resistant cultivar. **b** Mesophyll cell structure of the infected resistant cultivar. **c** Mesophyll cell structure of the mock susceptible cultivar. **d** Mesophyll cell structure of the infected susceptible cultivar. **e** Nucleus of the mock resistant cultivar. **f** Nucleus of the infected resistant cultivar. **g** Nucleus of the mock susceptible cultivar. **h** Nucleus of the infected susceptible cultivar. **i** Chloroplast of the mock resistant cultivar. **j** Chloroplast of the infected resistant cultivar. **k** Chloroplast of the mock susceptible cultivar. **l** Chloroplast of the infected susceptible cultivar. The resistant cultivar was Mianyang 26/Yumai 47, and the susceptible cultivar was CU42. Black arrows in (**a**-**d**) indicate mesophyll cells, black arrows in (**e**-**h**) indicate leaf nuclei, and black arrows in (**i**-**l**) indicate leaf chloroplasts (N: Nucleus; CHl: Chloroplast; St: Starch granule; M: Mitochondrion; scale bar of (**a**-**d**) =20 μm; scale bar of (**e**-**l**) =1 μm)

In the mesophyll cells (Fig. 6), the cell space was larger in the inoculated plants of the resistant cultivar than in the mock resistant cultivar (Fig. 6a, b). The mesophyll cells were scattered and deformed in the inoculated susceptible cultivar compared to those in the mock susceptible cultivar (Fig. 6c, d). Similarly, there were no significant differences between the nuclei of the inoculated resistant plants and those of the mock resistant plants (Fig. 6e, f). By contrast, the nuclear envelope was ruptured in the inoculated susceptible cultivar but not in the mock susceptible cultivar (Fig. 6g, h). We also found that the lamellar structure of the chloroplasts lost its rigidity and was deformed compared to that in the mock susceptible plants (Fig. 6k, l). However, few damaged chloroplasts were found in resistant cultivar or the mock treatment (Fig. 6i, j). All the above results indicated that *T. controversa* severely infects the cell tissues of the susceptible cultivar but not those of the resistant cultivar. The morphology of the roots, stems and leaves of the resistant and susceptible cultivars was also examined at the seedling growth stage (Z13) using transmission electron microscopy. However, smaller changes in the root, stem and leaf structures were found during the seedling growth stage (Z13) than during the tillering stage (Z21) (Additional files 13, 14 and 15). We concluded that the pathogen infected the susceptible cultivar much more severely at the tillering stage (Z21) than at the seedling growth stage (Z13).

Another resistant (Yinong 18/ Lankao 8) and susceptible cultivar (Dongxuan 3) were also used in this study. At the tillering stage (Z21) in the infected resistant cultivar, the changes in the roots, stems and leaves did not display obvious differences (Additional files 16a, b; e, f, i, j; 17a, b, e, f, i, j; 18a, b, e, f, i, j). However, the parenchyma cells in the root cortex of the susceptible cultivar (Dongxuan 3) were severely deformed compared to the mock treatment (Additional file 16c, d), and the root contents (Additional file 16g, h) and the nucleus (Additional file 16k, l) were all severely degraded. Stem cells from infected plants were arranged irregularly compared to the mock treatment (Additional file 17c, d), and the nuclei (Additional file 17g, h), and chloroplasts (Additional file 17k, l) showed severe deformations compared to the mock treatment. In the leaf, the mesophyll cells (Additional file 18c, d), nucleus (Additional file 18g, h) and chloroplast (Additional file 18k, l) were all severely deformed compared to the mock treatment. We also examined the root (Additional file 19), stem (Additional file 20) and leaf (Additional file 21) cells at the seedling growth stage (Z13). We found that *T. controversa* played a much more important role in the infected susceptible cultivar at the tillering stage (Z21) than at the seedling growth stage (Z13).

### Statistical analysis of the damaged critical corresponding cells in roots, stems, and leaves

For the seedling growth stage (Z13) and the tillering stage (Z21), we observed 100 ~ 400 critical corresponding cells from the roots, stems and leaves respectively by scanning electron microscopy and observed 40 ~ 120 critical corresponding cells separately in roots, stems and leaves by transmission electron microscopy. The percentages of damaged critical corresponding cells (the number of damaged critical corresponding cells /the total number of observed critical corresponding cells × 100%) were analyzed at the seedling growth stage (Z13) and tillering stage (Z21). Both results indicated that in the infected susceptible cultivars, the rate of damaged critical corresponding cells was significantly different between the two stages of wheat growth (Z13 and Z21) with *P*-values of 0.031, 0.008, 0.0097, 0.012, and 0.004 by scanning electron microscopy and 0.0277, 0.009, 0.0075, and 0.0224 by transmission electron microscopy (Table 1).

**Table 1:** Analysis of damaged critical corresponding cells in roots, stems, and leaves at the seedling growth stage (Z13) and tillering stage (Z21) in infected both wheat cultivars determined by scanning electron microscopy and transmission electron microscopy

Treatments	Total cells		Damaged cells		Rate of damaged cells (%)		*P*-value
	Z13	Z21	Z13	Z21	Z13	Z21	
Scanning electron microscopy							
S1-root	317	263	98	149	32.48 ± 9.55	56.85 ± 3.22	0.0031 *
S2-root	207	367	39	161	19.42 ± 2.55	44.58 ± 4.48	0.008 *
R1-root	394	390	47	41	11.74 ± 1.69	10.51 ± 0.52	0.5258
R2-root	319	377	23	29	7.34 ± 1.57	8.24 ± 3.34	0.819
S1-stem	102	184	24	62	23.50 ± 3.26	33.07 ± 2.08	0.0097 *
S2-stem	126	154	22	48	17.30 ± 1.49	31.06 ± 2.80	0.012 *
R1-stem	164	131	22	16	12.16 ± 3.63	11.91 ± 3.38	0.9617
R2-stem	101	106	7	6	7.37 ± 0.10	5.86 ± 1.73	0.488
S1-leaf	120	115	18	47	15.16 ± 1.14	41.21 ± 4.57	0.004 *
S2-leaf	107	126	38	60	37.14 ± 6.33	47.71 ± 1.27	0.177
R1-leaf	108	112	12	11	12.05 ± 3.28	10.50 ± 2.13	0.7102
R2-leaf	109	118	6	12	5.39 ± 0.09	10.12 ± 0.63	0.012
Transmission electron microscopy							
S1-root	45	137	17	59	36.36 ± 2.56	44.45 ± 5.91	0.0277 *
S2-root	21	37	7	13	30.45 ± 3.66	37.92 ± 4.10	0.246
R1-root	114	123	13	12	11.49 ± 0.71	9.95 ± 1.99	0.5073
R2-root	70	28	8	7	13.10 ± 2.10	26.33 ± 1.86	0.009 *
S1-stem	42	44	8	17	19.57 ± 2.20	39.88 ± 3.42	0.0075 *
S2-stem	27	23	5	6	19.53 ± 2.97	29.75 ± 4.49	0.130
R1-stem	56	58	4	9	7.11 ± 1.08	14.97 ± 1.54	0.14
R2-stem	41	44	6	7	14.11 ± 0.87	16.41 ± 1.01	0.160
S1-leaf	56	31	8	10	15.79 ± 3.88	34.81 ± 3.55	0.0224 *
S2-leaf	43	26	6	9	13.14 ± 0.43	38.79 ± 10.96	0.080
R1-leaf	50	34	3	3	7.04 ± 1.03	8.09 ± 1.97	0.6615
R2-leaf	74	25	5	4	7.93 ± 1.59	15.19 ± 2.28	0.059

Each entry in the table indicates the average cells, with total cells based on 100 ~ 400 critical corresponding cells by scanning electron microscopy and 4 ~ 120 critical corresponding cells by transmission electron microscopy. The percentages of damaged critical corresponding cells = the number of damaged critical corresponding cells**/**the total number of observed critical corresponding cells × 100%. The significant *P*-values were indicated as “*” for a significance level of 0.05 according to ANOVA (Duncan’s multiple range test). *S-root* indicates root cells in the susceptible wheat cultivar, *R-root* indicates root cells in the resistant wheat cultivar, *S-stem* indicates stem cells in the susceptible wheat cultivar, *R-stem* indicates stem cells in the resistant wheat cultivar, *S-leaf* indicates leaf cells in the susceptible wheat cultivar, and *R-leaf* indicates leaf cells in the resistant wheat cultivar. S1, S2, R1, and R2 are CU42, Dongxuan 3, Mianyang 26/Yumai 47, and Yinong 18/Lankao 8, respectively

## Discussion

In this study, we characterized the histological changes at both the tillering stage (Z21) and the seedling growth stage (Z13) in resistant and susceptible wheat plants infected by *T. controversa* using transmission electron microscopy and scanning electron microscopy, and these changes will play an important role in exploring the fungus-host interaction mechanism.

To determine the relative importance of the tillering stage (Z21) and the seedling growth stage (Z13), based on the combination of images of disease symptoms (Additional file 1), microscopic observation and molecular detection of the infected wheat cultivars (Additional files 2 and 3), we obtained samples for observation by scanning electron microscopy and transmission electron microscopy. According to the results, the plant histological characteristics at the tillering stage (Z21) were much more affected by the pathogen infection. Specifically, in the root cells at the tillering stage (Z21), scanning electron microscopy showed fungal hyphae in both vascular bundle cells and cortical parenchyma cells of the susceptible cultivars (CU42, Dongxuan 3) and the cortical parenchyma cells in one of the resistant wheat cultivars (Mianyang 26/Yumai 47). Additionally, the morphophysiological characteristics of the roots of infected susceptible plants were significantly deformed compared with those of the mock plants (Fig. 1, Additional file 7). At the seedling growth stage (Z13), we observed few changes by scanning electron microscopy (Additional files 4 and 10). With transmission electron microscopy, for the root cells, we observed cell deformation in the infected susceptible cultivar plants (Fig. 4, Additional files 13, 16 and 19). We also found that the root nucleus and the cell wall of nuclear degraded and ruptured in the inoculated susceptible cultivar; these changes were also found at the seedling growth stage (Z13), but the degree of damage was lower than that at the tillering stage (Z21). For the stem cells, we analyzed the results of the tillering stage (Z21) and the seedling growth stage (Z13) and found that the fungus successfully colonized, ruptured, and deformed the stem cells in the inoculated susceptible cultivar (Fig. 2, Additional file 8). In addition, the chloroplasts were degraded and ruptured in the inoculated susceptible cultivar but not in the mock plants at the tillering stage (Z21) (Fig. 5, Additional file 17); this change was not found at the seedling growth stage (Z13) (Additional files 5, 11, 14 and 20). In the leaf cells, even we found little changes in the cells with scanning electron microscopy in the infected susceptible cultivar (Fig. 3, Additional files 6, 9 and 12); while based on transmission electron microscopy, we observed that the nuclear envelopes were ruptured in the inoculated susceptible cultivar at the tillering stage (Z21), and we also found major changes in the lamella structure of the chloroplasts, which lost its rigidity and was deformed compared to that at the seedling growth stage (Z13) (Fig. 6, Additional files 15, 18 and 21).

In this study, we combined transmission electron microscopy and scanning electron microscopy to analyze the histological characteristics of root, stem, and leaf cells. Based on scanning electron microscopy, the stem and leaf cells were not highly different between the tillering stage (Z 21) (Figs. 2 and 3, Additional files 8 and 9) and the seedling growth stage (Z13) (Additional files 5, 6, 11 and 12). However, major differences were observed between the tillering stage (Z21) (Figs. 5 and 6, Additional files 17 and 18) and the seedling growth stage (Z13) (Additional files 14, 15, 20 and 21) by transmission electron microscopy. Therefore, it is essential and important to explore the histological changes using the combination of transmission electron and scanning electron microscopy.

Moreover, we found that *T. controversa* severely affected some cell organelles in the susceptible cultivar. For example, the fungal pathogen damaged the mesophyll cells of the susceptible cultivars and ruptured their cell structures (Fig. 6, Additional file 18). Other studies also observed similar phenomena in other crops due to different pathogens [25, 26]. Some reports mentioned that structural alterations were found in the host tissue of resistant plants, including the formation of gel plugs [27] or tyloses [27, 28] and the presence of substances in the xylem vessels [29, 30]. Leaf mesophyll cells play an important role in the photosynthesis process, which is also a key part of resistance to fungal pathogen invasion and expansion [31]. The cell nucleus is another organelle that changes in response to fungal infection [26]. Fungal pathogens can destroy the nuclear structures in plants after interacting with susceptible plants and change the chromatin condensation forms [32, 33]. Disorders in the chloroplast lamellae cause necrotic symptoms in plants and affect the average crop yields (Fig. 6, Additional files 15, 18 and 21). Chloroplasts are essential elements of photosynthesis, and chloroplast lamella infection reduces the plant height and average crop yield [34]. Ruptured chloroplast walls were observed by transmission electron microscopy in the inoculated susceptible cultivar (Fig. 5). Similar observations were observed in wheat infected with *Mycosphaerella graminicola* and *Puccinia striiformis* f. sp. *tritici* [26, 35]. Additionally, chloroplast content has been found to decrease after fungal infection [36]. The results revealed that the nuclei and chloroplasts in the roots, stems and leaves were intensively infected by *T. controversa* in the susceptible cultivar but not in the resistant plants (Figs. 4, 5 and 6). Mitochondria also play vital roles in cells and play crucial roles in the early defense responses of muskmelons against *Trichothecium roseum* infection through the regulation of ROS production and energy metabolism [37]. This finding suggests that mitochondria are very important in plant defense mechanisms. Our results showed that *T. controversa* dramatically affected the mitochondria in the susceptible cultivar but not those in the resistant cultivar (Additional file 14i, j, k, l). All these changes indicate that *T. controversa* affects cell organs in susceptible cultivar plants and damages their organelles, which inhibits the normal function of the plant. Hyphae invaded the tissue cells of the susceptible cultivar, which led to cell wall breakage, cell content degradation, and organelle destruction. The number of intercellular hyphae in the resistant cultivar was lower than that in the susceptible cultivar, which may be due to a defense mechanism that prevents the expansion of the hyphae and ensures the integrity of the cells and organelles. For the statistical analysis, we found that the percentage of damaged critical corresponding cells in the susceptible wheat cultivar was significantly different between the two growth stages of Z13 and Z21 (Table 1).

Some studies have described the root cell response to specific fungi. For example, a study on watermelon seedling infection by *Fusarium oxysporum* f. sp. *niveum* [38] noted the histological responses of susceptible watermelon seedlings only; thus, it could not be determined whether there were any differences between susceptible and resistant plants. In our study, we analyzed both susceptible and resistant plants using mock and infected plants, and we did find differences between them. This type of information is very important for exploring the infection mechanism of *T. controversa* and contributes to the effective control of wheat bunt.

In summary, this study reported on the characteristics of histological changes in resistant and susceptible cultivars at the tillering stage (Z21) and seedling growth stage (Z13), which will contribute greatly to the exploration of the infection mechanism of *T. controversa* and the interaction of this fungus with its host.

## Conclusions

The root, stem and leaf organelles of the susceptible cultivar were much more extensively affected by the fungal hyphae than those of the resistant plants at the tillering stage (Z21) than at the seedling growth stage (Z13).

## Methods

### Fungal material and culture

The identified fungal strain *T. controversa* was provided by Blair Goates, United States Department of Agriculture (USDA), Agricultural Research Service (ARS), Aberdeen, Idaho, USA. Plates containing *T. controversa* teliospores on 2% soil-agar media were incubated for 60 days under 24 h light (60 μmol/m^2^/s) at 5 °C in an incubator (MLR 352H, Panasonic, USA) after being covered with parafilm. The teliospore germination was observed under an automated inverted fluorescence microscope (IX83, Olympus, Japan). The hyphae were collected for inoculation at a concentration of 10^6^ spores/mL with an OD_600_ of 0.15 [39].

### Plant materials and growth conditions

Four winter wheat cultivars, Mianyang 26/Yumai 47, Yinong 18/ Lankao 8 as resistant cultivars and CU42, Dongxuan 3 as susceptible cultivars, were used in this experiment. The seeds were obtained from the Institute of Plant Protection, Chinese Academy of Agricultural Sciences. The seeds were surface-sterilized with 30% NaClO for 1 min, washed with sterile water 3 times and kept on plates on moist filter paper at 5 °C for 1 month to vernalize. Transplantation was performed when the wheat germ sheath grew to 1 ~ 3 cm (at the germination stage (Z09)). The seedlings were moved into pots filled with an organic matter and soil mix (Klasmann-Deilmann, Germany) at a ratio of 1:2% and grown in growth chambers (Percival, ARC-36VL-LT, USA). The wheat seedlings were grown under a 14 h light (300 μmol/m^2^/s)/10 h dark cycle at 8 °C during the seedling growth stage (Z11 ~ Z13) and at 15 °C during the tillering stage (Z21). At the seedling growth stage (Z11), the roots of every wheat seedling were injected with 2 mL of *T. controversa* inoculum suspension. The suspension contained infectious hyphae at a concentration of 10^6^ spores/ml with an OD_600_ of 0.15, and 2 mL of the suspension was injected next to the root with a syringe (5 mL) and needle gauge with diameter of 0.7 mm (Zhiyu, Jiangsu, China). Inoculation was repeated after 12 h, and the injections continued for 5 days. Plants grown under the same conditions were injected with sterilized ddH_2_O for use as the mock treatment. We established four treatments: mock resistant plants, mock susceptible plants, infected resistant plants, and infected susceptible plants. Each treatment included 30 wheat plants. For the successfully infected wheat plants, 20 plants of both resistant and susceptible cultivars were selected for later electron microscope observation. Similarly, for the mock wheat plants, 20 plants of both resistant and susceptible plants were selected.

### Detection of *T. controversa* in plants

For detection by microscopy, wheat germ agglutinin and Alexa Fluor 488 conjugate (WGA-AF 488) (Invitrogen, Eugene, USA) were used to stain the hyphae, and propidium iodide (PI) (Invitrogen, Eugene, USA) was used to stain the wheat tissues. The procedures were followed in accordance with Gao et al. [40]. The samples were observed under a confocal laser scanning microscope (Leica SP8, Germany) with excitation 510 nm/emission 570 nm (for WGA-488) and excitation 590/emission 680 nm (for PI).

For detection by molecular methods, genomic DNA was extracted from the wheat leaves using a plant genomic DNA kit (TianGen, Beijing, China). The sequence-characterized amplified region (SCAR) marker of *T. controversa* (ISSR859-140AF- 5′-TGGTGGTCGGGAAAGATTAGA-3, ISSR859-511AR: 5′-GGGACGAAGGCATCAAGAAG-3′) was used [41] in this study, and the primers were synthesized by a company (Takara, Beijing, China). The DNA concentration was 80 ng/μL. The PCR amplification conditions were as follows: initial denaturation at 94 °C for 5 min and 35 cycles of amplification with denaturation at 94 °C for 20 s, annealing at 56 °C for 20 s, and extension at 72 °C for 30 s with a final extension at 72 °C for 7 min. After the PCR, 2% agarose gel electrophoresis was performed at 150 V for 30 min, the gel was stained with ethidium bromide, and the expected bands were visualized using the gel documentation system (WSE-5200 Printgraph 2 M, ATTO, Korea). Successful infection was indicated by the positive amplification of the expected 372-bp band.

### Sample preparation for scanning electron microscopy

The roots, stems and leaves were collected from inoculated and mock plants of both cultivars. The plants were washed with ddH_2_O for further processing. A sterilized scalpel was used to cut leaf veins quickly, with the midrib as the axis of symmetry and a size of 5 mm × 5 mm [length × width]. Similarly, the roots and stems were cut to a length of approximately 5 mm. The pieces of roots, stems, and leaves were immediately placed in 3% glutaraldehyde for 48 h for staining. After staining, the samples were rinsed 10 times in phosphate buffer (0.1 mmol) and stored in osmium tetroxide (OsO_4_) (1%) for 1.5 h at room temperature (25 °C). The samples were dehydrated in a graded ethanol wash (30, 50, 60, 70, 80, 90 and 100%) for 20 min each. After being dehydrated, the samples were dried at 40 °C on a CO_2_ critical point dryer (Leica CPD 030, Germany) for 5 h. The samples were placed on conductive, double-sided adhesive tape to ensure that the samples did not move or fall and that the observed part of the sample remained at the same height. The samples were investigated by scanning electron microscopy (S-570, HITACHI, Japan) after spraying metal film onto the specimen surfaces as reported by Segado et al. [42].

### Sample preparation for transmission electron microscopy

The above method was also used for transmission electron microscopy, except vacuuming was required for dehydration. Once a specimen settled, it was immediately placed in the fixation solution and left for 48 h. After dehydration, the specimens were subjected to gradient infiltration with anhydrous acetone and embedded in epoxy resin. After being embedded, the samples were placed inside a dryer (Leica CPD 030, Germany) at 45 °C for 12 h and 60 °C for 48 h for polymerization in epoxy resin and then cut into ultrathin sections with an ultrathin slicer (Leica EM UC6, Germany). The sample was placed on an ultrathin slicer for cutting and double-stained with uranium acetate and lead citrate as described by Carisse et al. [16] and Xu et al. [43]. The samples were then observed using a transmission electron microscope (H-7650, HITACHI, Japan).

### Statistical analysis of the damaged critical corresponding cells in roots, stems, and leaves

The percentages of damaged critical corresponding cells in the roots, stems and leaves of *T. controversa*-infected resistant and susceptible cultivars at the seedling growth stage (Z13) and the tillering stage (Z21) were calculated. Twenty plants were selected as biological repeats. We performed the analyses with Statistical Analysis System version 6.10 (SAS Institute, Cary, NC) based on 100 ~ 400 critical corresponding cells by scanning electron microscopy and 4- ~ 120 critical corresponding cells by transmission electron microscopy. The *P*-values were indicated at a significance level of 0.05 according to the ANOVA (Duncan’s multiple range test).

## Supplementary Information

**Additional file 1: Fig. S1.** *T. controversa*-infected resistant and susceptible cultivars and the mock treatments at the seedling growth stage (Z13) and the tillering stage (Z21). (a) The mock susceptible cultivar at the seedling growth stage (Z13). (b) The *T. controversa*-infected susceptible cultivar at the seedling growth stage (Z13). (c) The mock resistant cultivar at the seedling growth stage (Z13). (d) The *T. controversa*-infected resistant cultivar at the seedling growth stage (Z13). (e) The mock susceptible cultivar at the tillering stage (Z21). (f) The *T. controversa*-infected susceptible cultivar at the tillering stage (Z21). (g) The mock resistant cultivar at the tillering stage (Z21). (h) The *T. controversa*-infected resistant cultivar at the tillering stage (Z21). The resistant cultivar was Mianyang 26/Yumai 47, and the susceptible cultivar was CU42.

**Additional file 2: Fig. S2.** The hyphae of *T. controversa* in the roots, stems and leaves of infected resistant and susceptible cultivars at the seedling growth stage (Z13) and tillering stage (Z21) under a laser scanning confocal microscope. (a)-(f) Indicate the seedling growth stage (Z13) and (g)-(l) indicate the tillering stage (Z21). (a) (g) *T. controversa*-infected roots of the resistant cultivar; (b) (h) *T. controversa*-infected stems of the resistant cultivar; (c) (i) *T. controversa*-infected leaves of the resistant cultivar; (d) (j) *T. controversa*-infected roots of the susceptible cultivar; (e) (k) *T. controversa*-infected stems of the susceptible cultivar; and (f) (l) *T. controversa*-infected leaves of the susceptible cultivar. The resistant cultivar was Mianyang 26/Yumai 47, and the susceptible cultivar was CU42. The red color indicates wheat tissues while the green color indicates the *T. controversa* hyphae.

**Additional file 3: Fig. S3.** Molecular detection of *T. controversa* in plants. M: 2000 DNA marker; 1: DNA from a *T. controversa* teliospore as the positive control; 2: DNA from mock resistant leaves (Z13); 3: DNA from mock resistant leaves (Z21); 4: DNA from mock susceptible leaves (Z13); 5: DNA from infected resistant leaves (Z13); 6: DNA from mock susceptible leaves (Z21); 7: DNA from infected susceptible leaves (Z13); 8: DNA from mock resistant leaves (Z13); 9, 10, DNA from infected resistant leaves (Z21); 12: sterilized ddH_2_O as the negative control. The resistant cultivar was Mianyang 26/Yumai 47 and the susceptible cultivar was CU42.

**Additional file 4: Fig. S4.** Histological characteristics of the roots of the mock and infected resistant and susceptible cultivars at the seedling growth stage (Z13) under scanning electron microscopy. (a) Epidermal cells of the mock resistant cultivar. (b) Epidermal cells of the infected resistant cultivar. (c) Epidermal cells of the mock susceptible cultivar. (d) Epidermal cells of the infected susceptible cultivar. (e) Vascular bundle cells of the mock resistant cultivar. (f) Vascular bundle cells of the infected resistant cultivar. (g) Vascular bundle cells of the mock susceptible cultivar. (h) Vascular bundle cells of the infected susceptible cultivar. The resistant cultivar was Mianyang 26/Yumai 47 and the susceptible cultivar was CU42. White arrows in (a)(b)(c)(d) indicate epidermal cells, black arrows in (a)(b)(c)(d) indicate root hairs, and white arrows in (e)(f)(g)(h) indicate vascular bundle cells.

**Additional file 5: Fig. S5.** Histological characteristics of the stems of the mock and infected resistant and susceptible cultivars at the seedling growth stage (Z13) under scanning electron microscopy. (a) Stem cell structure of the mock resistant cultivar. (b) Stem cell structure of the infected resistant cultivar. (c) Stem cell structure of the mock susceptible cultivar. (d) Stem cell structure of the infected susceptible cultivar. (e) Longitudinal section of the stem of the mock resistant cultivar. (f) Longitudinal section of the stem of the infected resistant cultivar. (g) Longitudinal section of the stem of the mock susceptible cultivar. (h) Longitudinal section of the stem of the infected susceptible cultivar. The resistant cultivar was Mianyang 26/Yumai 47, and the susceptible cultivar was CU42. White arrows in (a)(b)(c)(d) indicate stem cells and white arrows in (e)(f)(g)(h) indicate longitudinal section stem cells.

**Additional file 6: Fig. S6.** Histological characteristics of the leaves of the mock and infected resistant and susceptible cultivars at the seedling growth stage (Z13) under scanning electron microscopy. (a) Mesophyll cells of the mock resistant cultivar. (b) Mesophyll cells of the infected resistant cultivar. (c) Mesophyll cells of the mock susceptible cultivar. (d) Mesophyll cells of the infected susceptible cultivar. The resistant cultivar was Mianyang 26/Yumai 47 and the susceptible cultivar was CU42. White arrows in (a)(b)(c)(d) indicate mesophyll cells.

**Additional file 7: Fig. S7.** Histological characteristics of the roots of the mock and infected resistant and susceptible cultivars at the tillering stage (Z21) by scanning electron microscopy. (a) Vascular bundle cells and cortical parenchyma cells of the mock resistant cultivar. (b) Vascular bundle cells and cortical parenchyma cells of the infected resistant cultivar. (c) Vascular bundle cells and cortical parenchyma cells of the mock susceptible cultivar. (d) Vascular bundle cells and cortical parenchyma cells of the infected susceptible cultivar. (e) Root epidermal cells of the mock resistant cultivar. (f) Root epidermal cells of the infected resistant cultivar. (g) Root epidermal cells of the mock susceptible cultivar. (h) Root epidermal cells of the infected susceptible cultivar. The resistant cultivar was Yinong 18/Lankao 8 and the susceptible cultivar was Dongxuan 3. The white arrows in (a)(b)(c)(d) indicate cortical parenchyma cells, the black arrows in (a)(b)(c)(d) indicate vascular bundle cells and the white circles in (d) indicate hyphae in cortical parenchyma cells; the white arrows in (e)(f)(g)(h) indicate root epidermal cells.

**Additional file 8: Fig. S8.** Histological characteristics of the stems of the mock and infected resistant and susceptible cultivars at the tillering stage (Z21) under scanning electron microscopy. (a) Stem cell structure of the mock resistant cultivar. (b) Stem cell structure of the infected resistant cultivar. (c) Stem cell structure of the mock susceptible cultivar. (d) Stem cell structure of the infected susceptible cultivar. (e) Longitudinal section of the stem of the mock resistant cultivar. (f) Longitudinal section of the stem of the infected resistant cultivar. (g) Longitudinal section of the stem of the mock susceptible cultivar. (h) Longitudinal section of the stem of the infected susceptible cultivar. The resistant cultivar was Yinong 18/Lankao 8, and the susceptible cultivar was Dongxuan 3. White arrows in (a)(b)(c)(d) indicate stem cells and white arrows in (e)(f)(g)(h) indicate longitudinal section stem cells.

**Additional file 9: Fig. S9.** Histological characteristics of the leaves of the mock and infected resistant and susceptible cultivars at the tillering stage (Z21) under scanning electron microscopy. (a) Mesophyll cells of the mock resistant cultivar. (b) Mesophyll cells of the infected resistant cultivar. (c) Mesophyll cells of the mock susceptible cultivar. (d) Mesophyll cells of the infected susceptible cultivar. The resistant cultivar was Yinong 18/Lankao 8, and the susceptible cultivar was Dongxuan 3. White arrows in (a)(b)(c)(d) indicate mesophyll cells.

**Additional file 10: Fig. S10.** Histological characteristics of the roots of the mock and infected resistant and susceptible cultivars at the seedling growth stage (Z13) under scanning electron microscopy. (a) Vascular bundle cells and cortical parenchyma cells of the mock resistant cultivar. (b) Vascular bundle cells and cortical parenchyma cells of the infected resistant cultivar. (c) Vascular bundle cells and cortical parenchyma cells of the mock susceptible cultivar. (d) Vascular bundle cells and cortical parenchyma cells of the infected susceptible cultivar. (e) Root epidermal cells of the mock resistant cultivar. (f) Root epidermal cells of the infected resistant cultivar. (g) Root epidermal cells of the mock susceptible cultivar. (h) Root epidermal cells of the infected susceptible cultivar. The resistant cultivar was Yinong 18/Lankao 8, and the susceptible cultivar was Dongxuan 3. White arrows in (a)(b)(c)(d) indicate cortical parenchyma cells, black arrows in (a)(b)(c)(d) indicate vascular bundle cells and white arrows in (e)(f)(g)(h) indicate root epidermal cells.

**Additional file 11: Fig. S11.** Histological characteristics of the stems of the mock and infected resistant and susceptible cultivars at the seedling growth stage (Z13) under scanning electron microscopy. (a) Stem cell structure of the mock resistant cultivar. (b) Stem cell structure of the infected resistant cultivar. (c) Stem cell structure of the mock susceptible cultivar. (d) Stem cell structure of the infected susceptible cultivar. The resistant cultivar was Yinong 18/Lankao 8, and the susceptible cultivar was Dongxuan 3. White arrows in (a)(b)(c)(d) indicate stem cells.

**Additional file 12: Fig. S12.** Histological characteristics of the leaves of the mock and infected resistant and susceptible cultivars at the seedling growth stage (Z13) under scanning electron microscopy. (a) Mesophyll cells of the mock resistant cultivar. (b) Mesophyll cells of the infected resistant cultivar. (c) Mesophyll cells of the mock susceptible cultivar. (d) Mesophyll cells of the infected susceptible cultivar. The resistant cultivar was Yinong 18/Lankao 8, and the susceptible cultivar was Dongxuan 3. White arrows in (a)(b)(c)(d) indicate mesophyll cells.

**Additional file 13: Fig. 13.** Histological characteristics of the roots of the mock and infected resistant and susceptible cultivars at the seedling growth stage (Z13) under transmission electron microscopy. (a) Vascular bundle cells of the mock resistant cultivar. (b) Vascular bundle cells of the infected resistant cultivar. (c) Vascular bundle cells of the mock susceptible cultivar. (d) Vascular bundle cells of the infected susceptible cultivar. (e) Root nucleus of the mock resistant cultivar. (f) Root nucleus of the infected resistant cultivar. (g) Root nucleus of the mock susceptible cultivar. (h) Root nucleus of the infected susceptible cultivar. The resistant cultivar was Mianyang 26/Yumai 47, and the susceptible cultivar was CU42. Black arrows in (a)(b)(c)(d) indicate vascular bundle cells, and black arrows in (e)(f)(g)(h) indicate root nucleus. (N: Nucleus; M: Mitochondrion; scale bar of (a)(b)(c)(d) =10 μm; scale bar of (e)(f) =1 μm; scale bar of (g)(h) =2 μm).

**Additional file 14: Fig. S14.** Histological characteristics of stem cells of the mock and infected resistant and susceptible cultivars at the seedling growth stage (Z13) under transmission electron microscopy. (a) Stem cell structure of the mock resistant cultivar. (b) Stem cell structure of the infected resistant cultivar. (c) Stem cell structure of the mock susceptible cultivar. (d) Stem cell structure of the infected susceptible cultivar. (e) Stem cell nucleus of the mock resistant cultivar. (f) Stem cell nucleus of the infected resistant cultivar. (g) Stem cell nucleus of the mock susceptible cultivar. (h) Stem cell nucleus of the infected susceptible cultivar. (i) Stem mitochondrion of the mock resistant cultivar. (j) Stem mitochondrion of the infected resistant cultivar. (k) Stem mitochondria of the mock susceptible cultivar. (l) Stem mitochondrion of the infected susceptible cultivar. The resistant cultivar was Mianyang 26/Yumai 47, and the susceptible cultivar was CU42. Black arrows in (a)(b)(c)(d) indicate stem cells, black arrows in (e)(f)(g)(h) indicate stem cell nucleus, and black arrows in (i)(j)(k)(l) indicate stem mitochondrion. (N: Nucleus; M: Mitochondrion; scale bar of (a)(b)(c)(d) =10 μm; scale bar of (e)(f)(g)(h) =1 μm; scale bar of (i)(j)(k)(l) =0.5 μm).

**Additional file 15: Fig. S15.** Histological characteristics of the leaves of the mock and infected resistant and susceptible cultivars at the seedling growth stage (Z13) under transmission electron microscopy. (a) Mesophyll cell structure of mock resistant plants. (b) Mesophyll cell structure of infected resistant plants. (c) Mesophyll cell structure of mock susceptible plants. (d) Mesophyll cell structure of infected susceptible plants. (e) Nucleus of mock resistant plants. (f) Nucleus of infected resistant plants. (g) Nucleus of mock susceptible plants. (h) Nucleus of infected susceptible plants. (i) Chloroplast of mock resistant plants. (j) Chloroplast of infected resistant plants. (k) Chloroplast of mock susceptible plants. (l) Chloroplast of infected susceptible plants. The resistant cultivar was Mianyang 26/Yumai 47 and the susceptible cultivar was CU42. Black arrows in (a)(b)(c)(d) indicate mesophyll cells, black arrows in (e)(f)(g)(h) indicate leaf nucleus, black arrows in (a)(b)(c)(d) indicate stem cells, and black arrows in (i)(j)(k)(l) indicate leaf chloroplasts (N: Nucleus; CHl: Chloroplast; St: Starch granule; M: Mitochondrion; scale bar of (a)(b)(c)(d) =10 μm; scale bar of (e)(f)(g)(h) =1 μm; scale bar of (i)(j)(k)(l) =0.5 μm).

**Additional file 16: Fig. S16.** Histological characteristics of the roots of the mock and infected resistant and susceptible cultivars at the tillering stage (Z21) under transmission electron microscopy. (a) Vascular bundle cells and cortical parenchyma cells of the mock resistant cultivar. (b) Vascular bundle cells and cortical parenchyma cells of the infected resistant cultivar. (c) Vascular bundle cells and cortical parenchyma cells of the mock susceptible cultivar. (d) Vascular bundle cells and cortical parenchyma cells of the infected susceptible cultivar. (e) Root cell contents of the mock resistant cultivar. (f) Root cell contents of the infected resistant cultivar. (g) Root cell contents of the mock susceptible cultivar. (h) Root cell contents of the infected susceptible cultivar. (i) Root nucleus of the mock resistant cultivar. (j) Root nucleus of the infected resistant cultivar. (k) Root nucleus of the mock susceptible cultivar. (l) Root nucleus of the infected susceptible cultivar. The resistant cultivar was Yinong 18/Lankao8 and the susceptible cultivar was Dongxuan 3. Black arrows in (a)(b)(c)(d) indicate cortical parenchyma cells, red arrows in (a)(b)(c)(d) indicate vascular bundle cells, black arrows in (e)(f)(g)(h) indicate root cell contents, and black arrows in (i)(j)(k)(l) indicate root nuclei (N: Nucleus; M: Mitochondrion; scale bar of (a)(b)(c)(d)(g) = 20 μm; scale bar of (e)(f) (h) =10 μm; scale bar of (i)(j) (k)(l) =1 μm).

**Additional file 17: Fig. S17.** Histological characteristics of the stems of the mock and infected resistant and susceptible cultivars at the tillering stage (Z21) under transmission electron microscopy. (a) Stem cell structure of the mock resistant cultivar. (b) Stem cell structure of the infected resistant cultivar. (c) Stem cell structure of the mock susceptible cultivar. (d) Stem cell structure of the infected susceptible cultivar. (e) Stem cell nucleus of the mock resistant cultivar. (f) Stem cell nucleus of the infected resistant cultivar. (g) Stem cell nucleus of the mock susceptible cultivar. (h) Stem cell nucleus of the infected susceptible cultivar. (i) Stem chloroplast of the mock resistant cultivar. (j) Stem chloroplast of the infected resistant cultivar. (k) Stem chloroplast of the mock susceptible cultivar. (l) Stem chloroplast of the infected susceptible cultivar. The resistant cultivar was Yinong 18/Lankao 8, and the susceptible cultivar was Dongxuan 3. Black arrows in (a)(b)(c)(d) indicate stem cells, black arrows in (e)(f)(g)(h) indicate stem nuclei, and black arrows in (i)(j)(k)(l) indicate stem chloroplasts (N: Nuclei; CHl: Chloroplast; St: Starch granule; scale bar of (a)(g)(h)(j) (l) =2 μm; scale bar of (b)(c)(d) = 5 μm; scale bar of (e)(f)(i) =1 μm; scale bar of (k) =0.5 μm).

**Additional file 18: Fig. S18.** Histological characteristics of the leaves of the mock and infected resistant and susceptible cultivars at the tillering stage (Z21) under transmission electron microscopy. (a) Mesophyll cell structure of the mock resistant cultivar. (b) Mesophyll cell structure of the infected resistant cultivar. (c) Mesophyll cell structure of the mock susceptible cultivar. (d) Mesophyll cell structure of the infected susceptible cultivar. (e) Nucleus of the mock resistant cultivar. (f) Nucleus of the infected resistant cultivar. (g) Nucleus of the mock susceptible cultivar. (h) Nucleus of the infected susceptible cultivar. (i) Chloroplast of the mock resistant cultivar. (j) Chloroplast of the infected resistant cultivar. (k) Chloroplast of the mock susceptible cultivar. (l) Chloroplast of the infected susceptible cultivar. The resistant cultivar was Yinong 18/Lankao 8, and the susceptible cultivar was Dongxuan 3. Black arrows in (a)(b)(c)(d) indicate mesophyll cells, black arrows in (e)(f)(g)(h) indicate leaf nuclei, and black arrows in (i)(j)(k)(l) indicate leaf chloroplasts (N: Nucleus; CHl: Chloroplast; St: Starch granule; M: Mitochondrion; scale bar of (a)(b) =10 μm; scale bar of (c)(d) =20 μm; scale bar of (e)(f)(g)(h)(i)(j)(k)(l) =2 μm).

**Additional file 19: Fig. S19.** Histological characteristics of the roots of the mock and infected resistant and susceptible cultivars at the seedling growth stage (Z13) under transmission electron microscopy. (a) Vascular bundle cells of the mock resistant cultivar. (b) Vascular bundle cells of the infected resistant cultivar. (c) Vascular bundle cells of the mock susceptible cultivar. (d) Vascular bundle cells of the infected susceptible cultivar. (e) Root nucleus of the mock resistant cultivar. (f) Root nucleus of the infected resistant cultivar. (g) Root nucleus of the mock susceptible cultivar. (h) Root nucleus of the infected susceptible cultivar. The resistant cultivar was Yinong 18/Lankao 8, and the susceptible cultivar was Dongxuan 3. Black arrows in (a)(b)(c)(d) indicate vascular bundle cells, and black arrows in (e)(f)(g)(h) indicate root nucleus. (N: Nucleus; M: Mitochondrion; scale bar of (a)(b) =20 μm; scale bar of (c)(d) =10 μm; scale bar of (e)(f) =1 μm; scale bar of (g)(h) =2 μm).

**Additional file 20: Fig. S20.** Histological characteristics of stem cells of the mock and infected resistant and susceptible cultivars at the seedling growth stage (Z13) under transmission electron microscopy. (a) Stem cell structure of the mock resistant cultivar. (b) Stem cell structure of the infected resistant cultivar. (c) Stem cell structure of the mock susceptible cultivar. (d) Stem cell structure of the infected susceptible cultivar. (e) Stem cell nucleus of the mock resistant cultivar. (f) Stem cell nucleus of the infected resistant cultivar. (g) Stem cell nucleus of the mock susceptible cultivar. (h) Stem cell nucleus of the infected susceptible cultivar. (i) Stem mitochondrion of the mock resistant cultivar. (j) Stem mitochondrion of the infected resistant cultivar. (k) Stem mitochondria of the mock susceptible cultivar. (l) Stem mitochondrion of the infected susceptible cultivar. The resistant cultivar was Yinong 18/Lankao 8, and the susceptible cultivar was Dongxuan 3. Black arrows in (a)(b)(c)(d) indicate stem cells, black arrows in (e)(f)(g)(h) indicate stem cell nucleus, and black arrows in (i)(j)(k)(l) indicate stem mitochondrion. (N: Nucleus; M: Mitochondrion; scale bar of (a)(b)(c)(d) =20 μm; scale bar of (e)(f)(g)(h) =2 μm; scale bar of (i)(j)(k)(l) =200 nm).

**Additional file 21: Fig. S21.** Histological characteristics of the leaves of the mock and infected resistant and susceptible cultivars at the seedling growth stage (Z13) under transmission electron microscopy. (a) Mesophyll cell structure of mock resistant plants. (b) Mesophyll cell structure of infected resistant plants. (c) Mesophyll cell structure of mock susceptible plants. (d) Mesophyll cell structure of infected susceptible plants. (e) Nucleus of mock resistant plants. (f) Nucleus of infected resistant plants. (g) Nucleus of mock susceptible plants. (h) Nucleus of infected susceptible plants. (i) Chloroplast of mock resistant plants. (j) Chloroplast of infected resistant plants. (k) Chloroplast of mock susceptible plants. (l) Chloroplast of infected susceptible plants. The resistant cultivar was Yinong 18/Lankao 8 and the susceptible cultivar was Dongxuan 3. Black arrows in (a)(b)(c)(d) indicate mesophyll cells, black arrows in (e)(f)(g)(h) indicate leaf nucleus, black arrows in (a)(b)(c)(d) indicate stem cells, and black arrows in (i)(j)(k)(l) indicate leaf chloroplasts. (N: Nucleus; CHl: Chloroplast; St: Starch granule; M: Mitochondrion; scale bar of (a)(b)(c)(d) =20 μm; scale bar of (e)(f) =2 μm; scale bar of (g)(h) =1 μm; scale bar of (i)(j) =0.5 μm; scale bar of (k)(l) =1 μm).

## Data Availability

All the data supporting our findings are contained within the manuscript.
