# Supplementary figures and images for: Characterization of histological changes at the tillering stage (Z21) in resistant and susceptible wheat plants infected by Tilletia controversa Kühn
Source: BMC Plant Biol. 2021 Jan 18;21:49. doi: 10.1186/s12870-020-02819-0 (PMC7814547; doi:10.1186/s12870-020-02819-0)

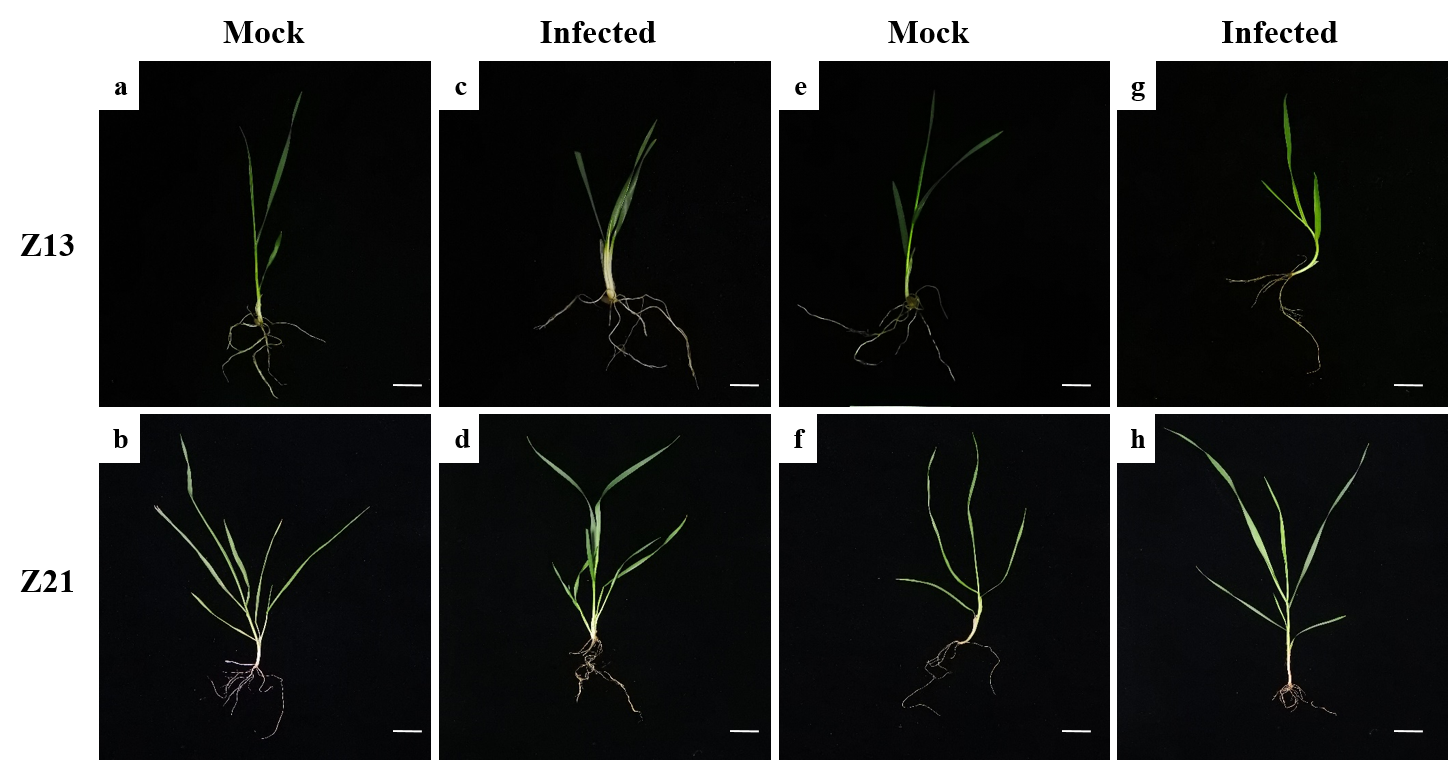

Supplement: Supplementary file 1 — Additional file 1: Fig. S1. T. controversa-infected resistant and susceptible cultivars and the mock treatments at the seedling growth stage (Z13) and the tillering stage (Z21). (a) The mock susceptible cultivar at the seedling growth stage (Z13). (b) The T. controversa-infected susceptible cultivar at the seedling growth stage (Z13). (c) The mock resistant cultivar at the seedling growth stage (Z13). (d) The T. controversa-infected resistant cultivar at the seedling growth stage (Z13). (e) The mock susceptible cultivar at the tillering stage (Z21). (f) The T. controversa-infected susceptible cultivar at the tillering stage (Z21). (g) The mock resistant cultivar at the tillering stage (Z21). (h) The T. controversa-infected resistant cultivar at the tillering stage (Z21). The resistant cultivar was Mianyang 26/Yumai 47, and the susceptible cultivar was CU42. [file 12870_2020_2819_MOESM1_ESM.tif]

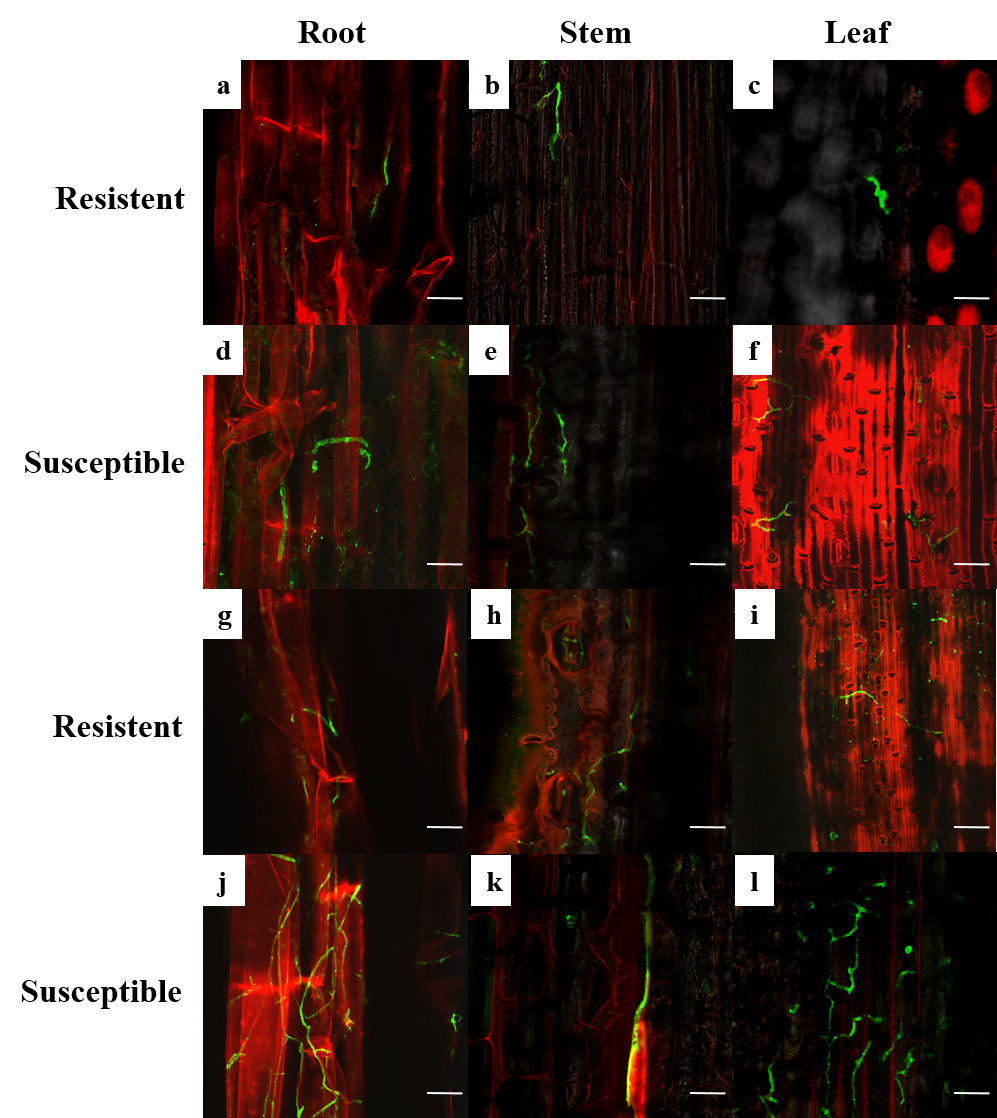

Supplement: Supplementary file 2 — Additional file 2: Fig. S2. The hyphae of T. controversa in the roots, stems and leaves of infected resistant and susceptible cultivars at the seedling growth stage (Z13) and tillering stage (Z21) under a laser scanning confocal microscope. (a)-(f) Indicate the seedling growth stage (Z13) and (g)-(l) indicate the tillering stage (Z21). (a) (g) T. controversa-infected roots of the resistant cultivar; (b) (h) T. controversa-infected stems of the resistant cultivar; (c) (i) T. controversa-infected leaves of the resistant cultivar; (d) (j) T. controversa-infected roots of the susceptible cultivar; (e) (k) T. controversa-infected stems of the susceptible cultivar; and (f) (l) T. controversa-infected leaves of the susceptible cultivar. The resistant cultivar was Mianyang 26/Yumai 47, and the susceptible cultivar was CU42. The red color indicates wheat tissues while the green color indicates the T. controversa hyphae. [file 12870_2020_2819_MOESM2_ESM.tif]

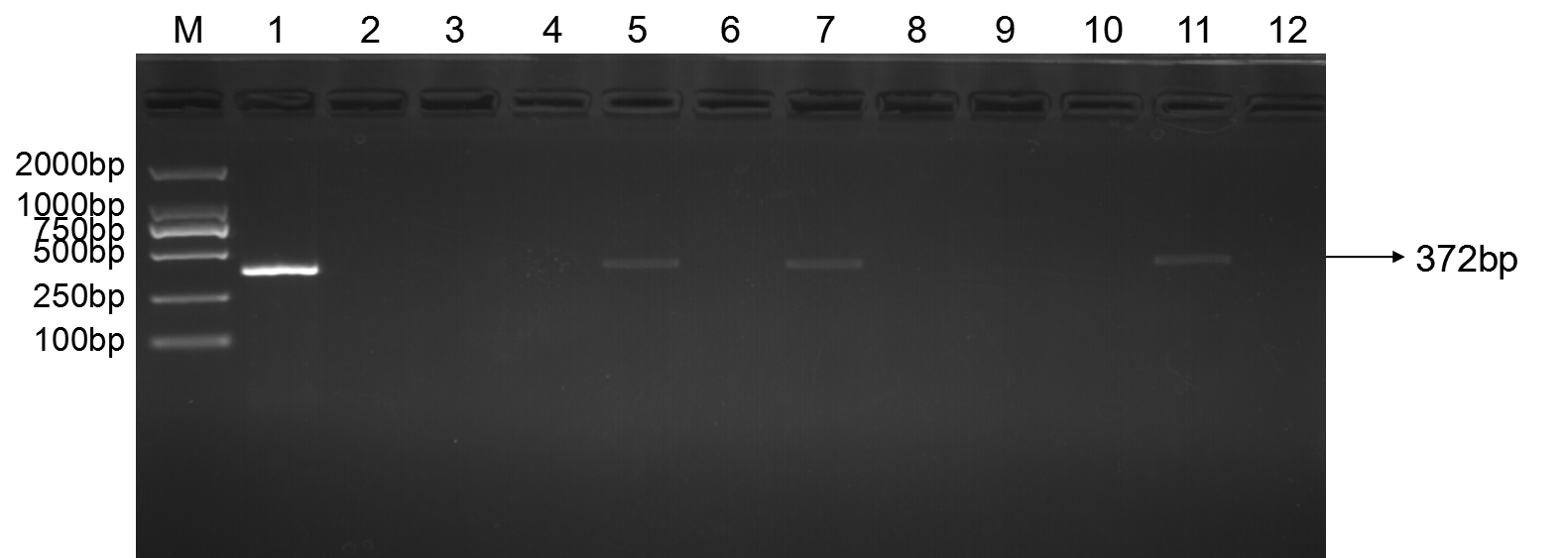

Supplement: Supplementary file 3 — Additional file 3: Fig. S3. Molecular detection of T. controversa in plants. M: 2000 DNA marker; 1: DNA from a T. controversa teliospore as the positive control; 2: DNA from mock resistant leaves (Z13); 3: DNA from mock resistant leaves (Z21); 4: DNA from mock susceptible leaves (Z13); 5: DNA from infected resistant leaves (Z13); 6: DNA from mock susceptible leaves (Z21); 7: DNA from infected susceptible leaves (Z13); 8: DNA from mock resistant leaves (Z13); 9, 10, DNA from infected resistant leaves (Z21); 12: sterilized ddH2O as the negative control. The resistant cultivar was Mianyang 26/Yumai 47 and the susceptible cultivar was CU42. [file 12870_2020_2819_MOESM3_ESM.tif]

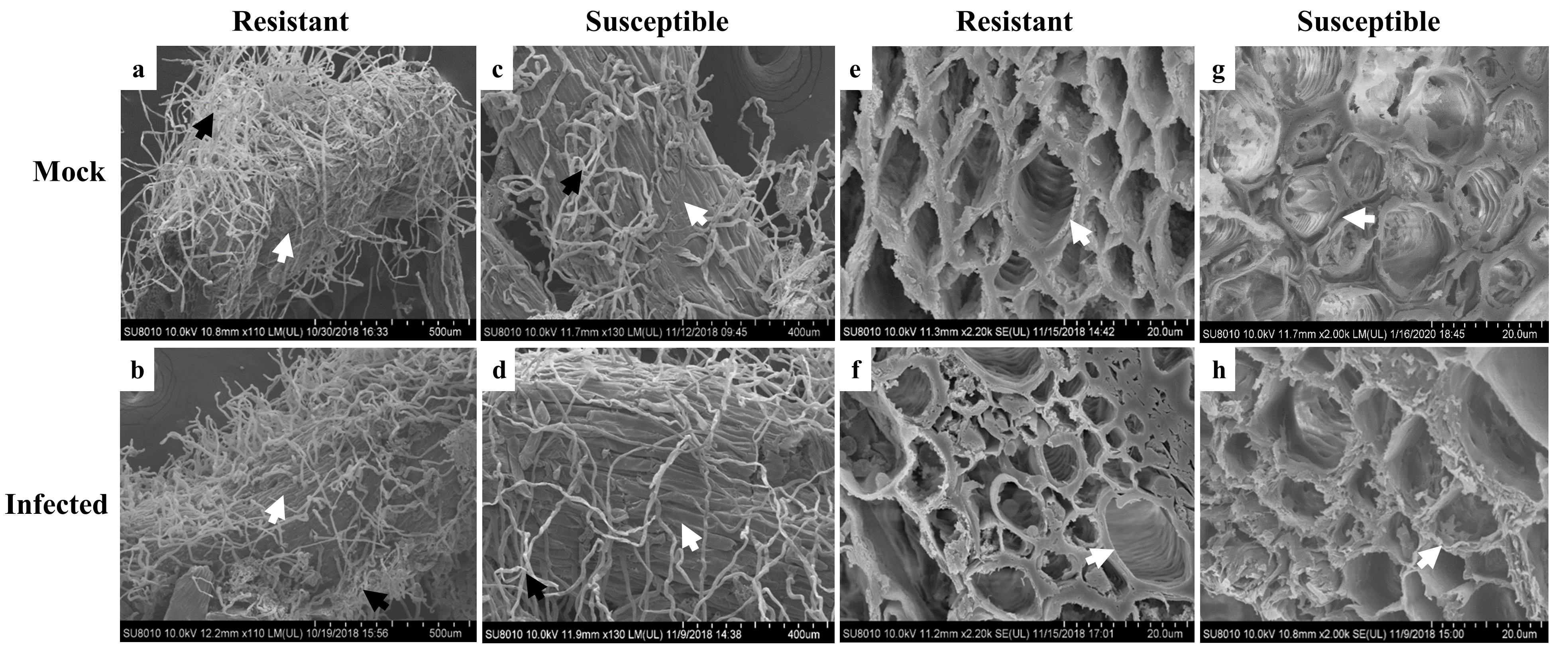

Supplement: Supplementary file 4 — Additional file 4: Fig. S4. Histological characteristics of the roots of the mock and infected resistant and susceptible cultivars at the seedling growth stage (Z13) under scanning electron microscopy. (a) Epidermal cells of the mock resistant cultivar. (b) Epidermal cells of the infected resistant cultivar. (c) Epidermal cells of the mock susceptible cultivar. (d) Epidermal cells of the infected susceptible cultivar. (e) Vascular bundle cells of the mock resistant cultivar. (f) Vascular bundle cells of the infected resistant cultivar. (g) Vascular bundle cells of the mock susceptible cultivar. (h) Vascular bundle cells of the infected susceptible cultivar. The resistant cultivar was Mianyang 26/Yumai 47 and the susceptible cultivar was CU42. White arrows in (a)(b)(c)(d) indicate epidermal cells, black arrows in (a)(b)(c)(d) indicate root hairs, and white arrows in (e)(f)(g)(h) indicate vascular bundle cells. [file 12870_2020_2819_MOESM4_ESM.tif]

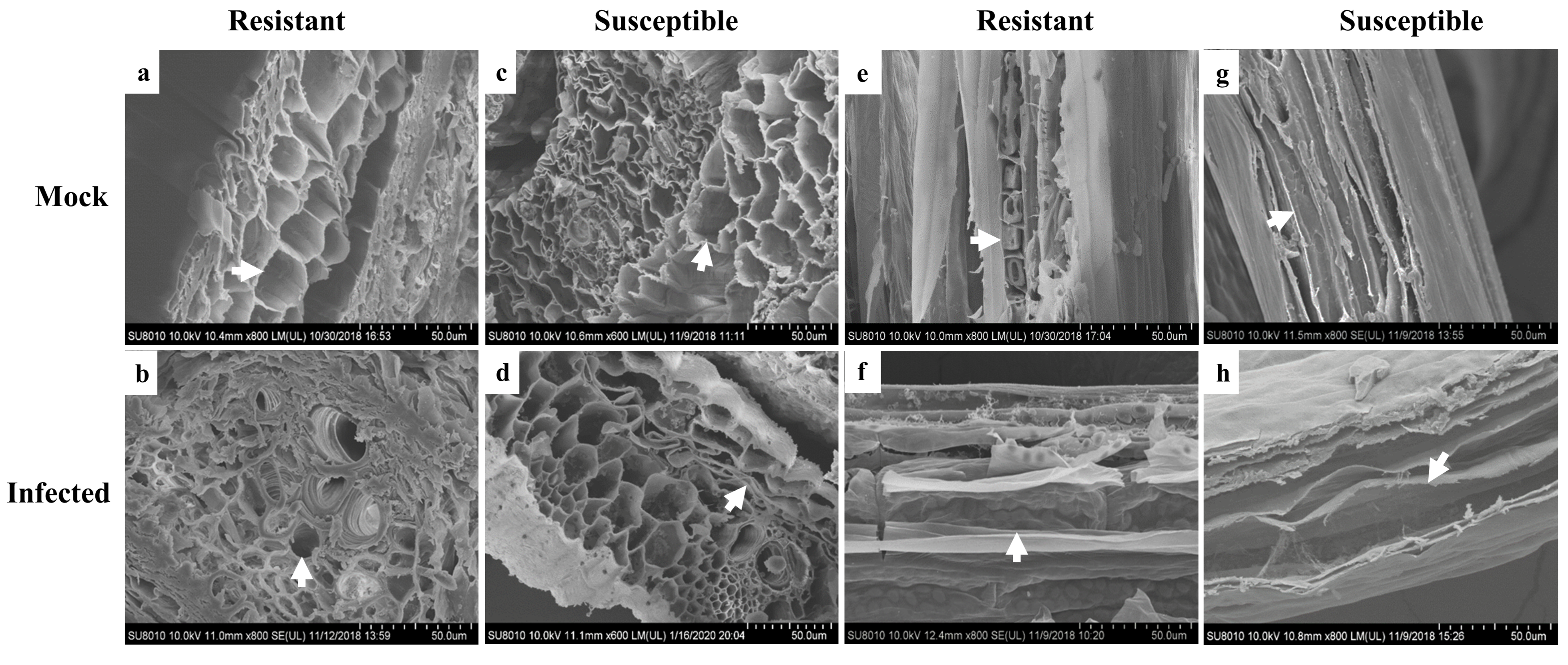

Supplement: Supplementary file 5 — Additional file 5: Fig. S5. Histological characteristics of the stems of the mock and infected resistant and susceptible cultivars at the seedling growth stage (Z13) under scanning electron microscopy. (a) Stem cell structure of the mock resistant cultivar. (b) Stem cell structure of the infected resistant cultivar. (c) Stem cell structure of the mock susceptible cultivar. (d) Stem cell structure of the infected susceptible cultivar. (e) Longitudinal section of the stem of the mock resistant cultivar. (f) Longitudinal section of the stem of the infected resistant cultivar. (g) Longitudinal section of the stem of the mock susceptible cultivar. (h) Longitudinal section of the stem of the infected susceptible cultivar. The resistant cultivar was Mianyang 26/Yumai 47, and the susceptible cultivar was CU42. White arrows in (a)(b)(c)(d) indicate stem cells and white arrows in (e)(f)(g)(h) indicate longitudinal section stem cells. [file 12870_2020_2819_MOESM5_ESM.tif]

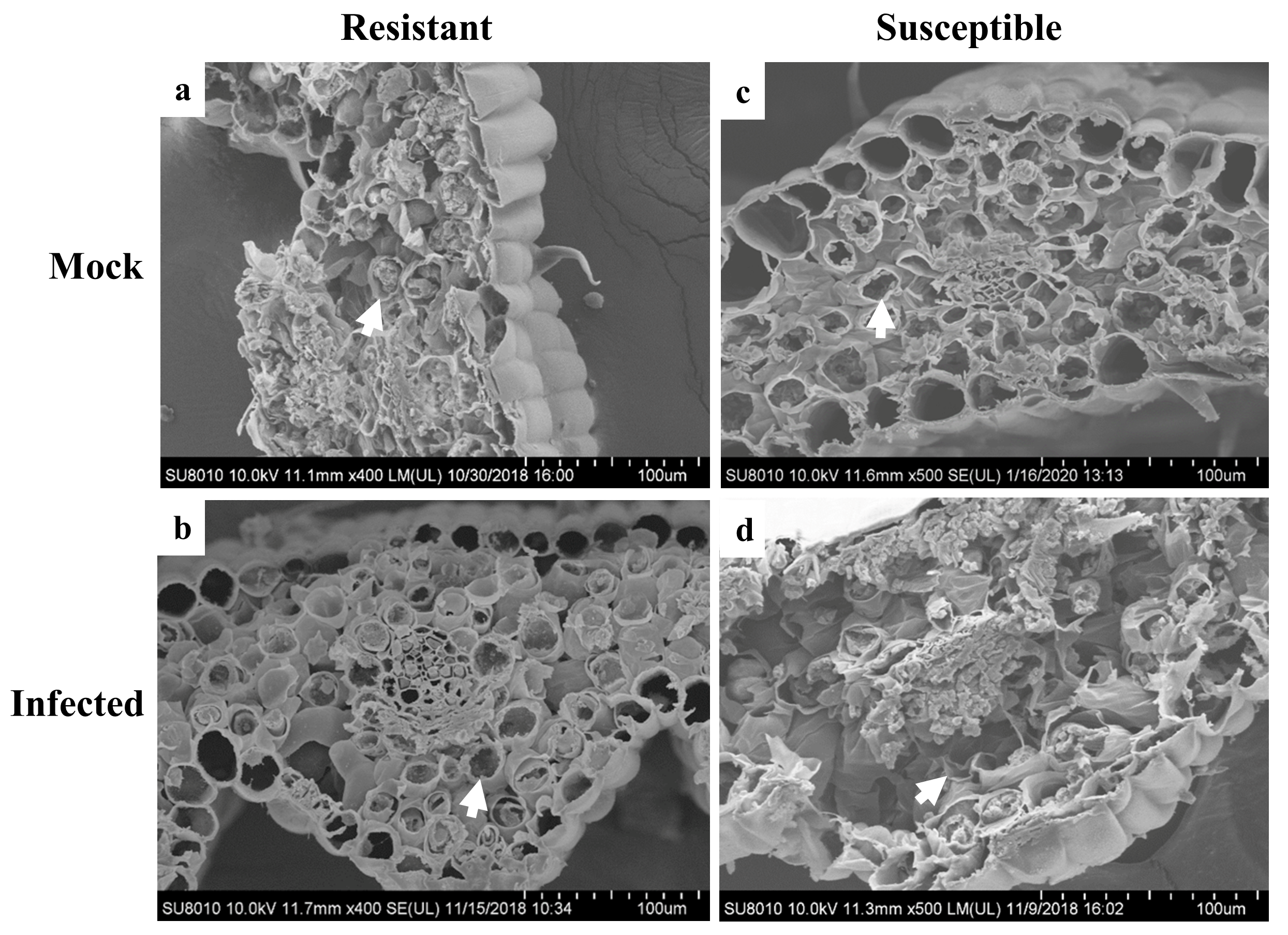

Supplement: Supplementary file 6 — Additional file 6: Fig. S6. Histological characteristics of the leaves of the mock and infected resistant and susceptible cultivars at the seedling growth stage (Z13) under scanning electron microscopy. (a) Mesophyll cells of the mock resistant cultivar. (b) Mesophyll cells of the infected resistant cultivar. (c) Mesophyll cells of the mock susceptible cultivar. (d) Mesophyll cells of the infected susceptible cultivar. The resistant cultivar was Mianyang 26/Yumai 47 and the susceptible cultivar was CU42. White arrows in (a)(b)(c)(d) indicate mesophyll cells. [file 12870_2020_2819_MOESM6_ESM.tif]

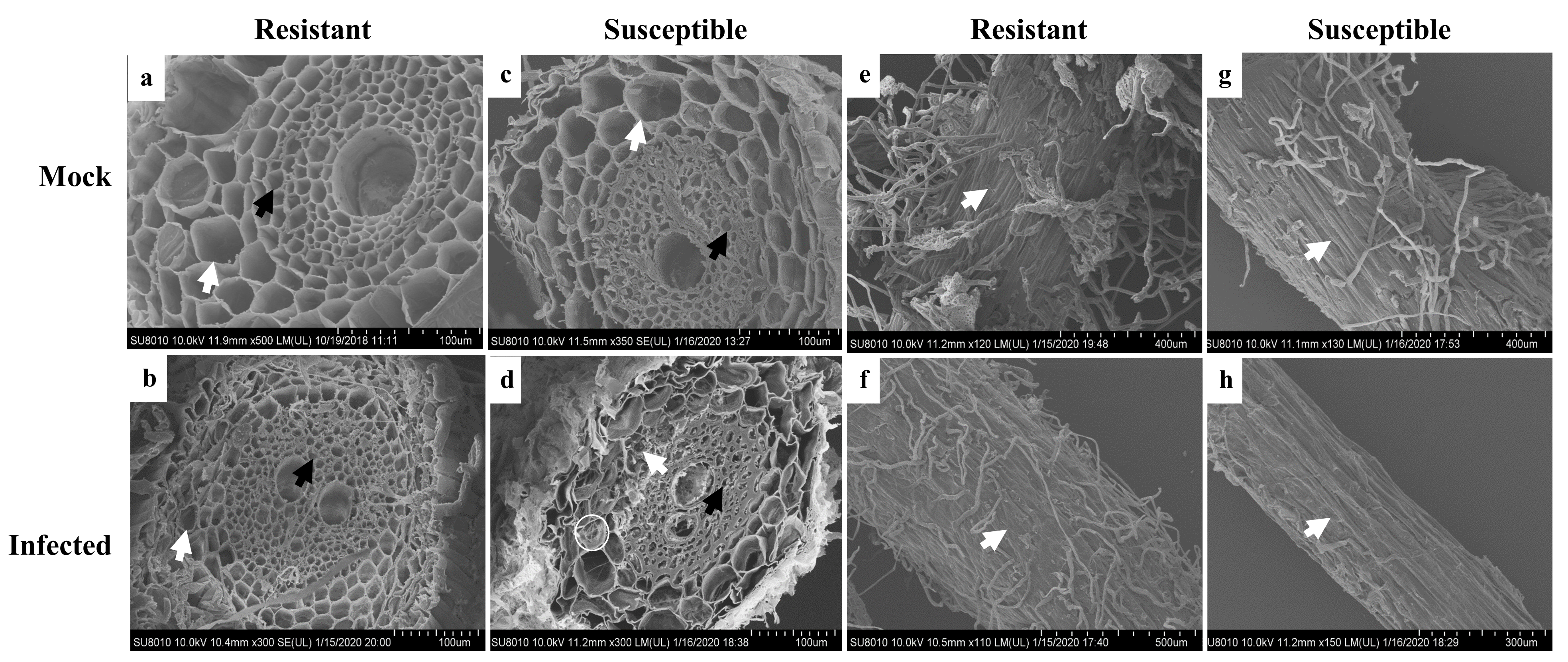

Supplement: Supplementary file 7 — Additional file 7: Fig. S7. Histological characteristics of the roots of the mock and infected resistant and susceptible cultivars at the tillering stage (Z21) by scanning electron microscopy. (a) Vascular bundle cells and cortical parenchyma cells of the mock resistant cultivar. (b) Vascular bundle cells and cortical parenchyma cells of the infected resistant cultivar. (c) Vascular bundle cells and cortical parenchyma cells of the mock susceptible cultivar. (d) Vascular bundle cells and cortical parenchyma cells of the infected susceptible cultivar. (e) Root epidermal cells of the mock resistant cultivar. (f) Root epidermal cells of the infected resistant cultivar. (g) Root epidermal cells of the mock susceptible cultivar. (h) Root epidermal cells of the infected susceptible cultivar. The resistant cultivar was Yinong 18/Lankao 8 and the susceptible cultivar was Dongxuan 3. The white arrows in (a)(b)(c)(d) indicate cortical parenchyma cells, the black arrows in (a)(b)(c)(d) indicate vascular bundle cells and the white circles in (d) indicate hyphae in cortical parenchyma cells; the white arrows in (e)(f)(g)(h) indicate root epidermal cells. [file 12870_2020_2819_MOESM7_ESM.tif]

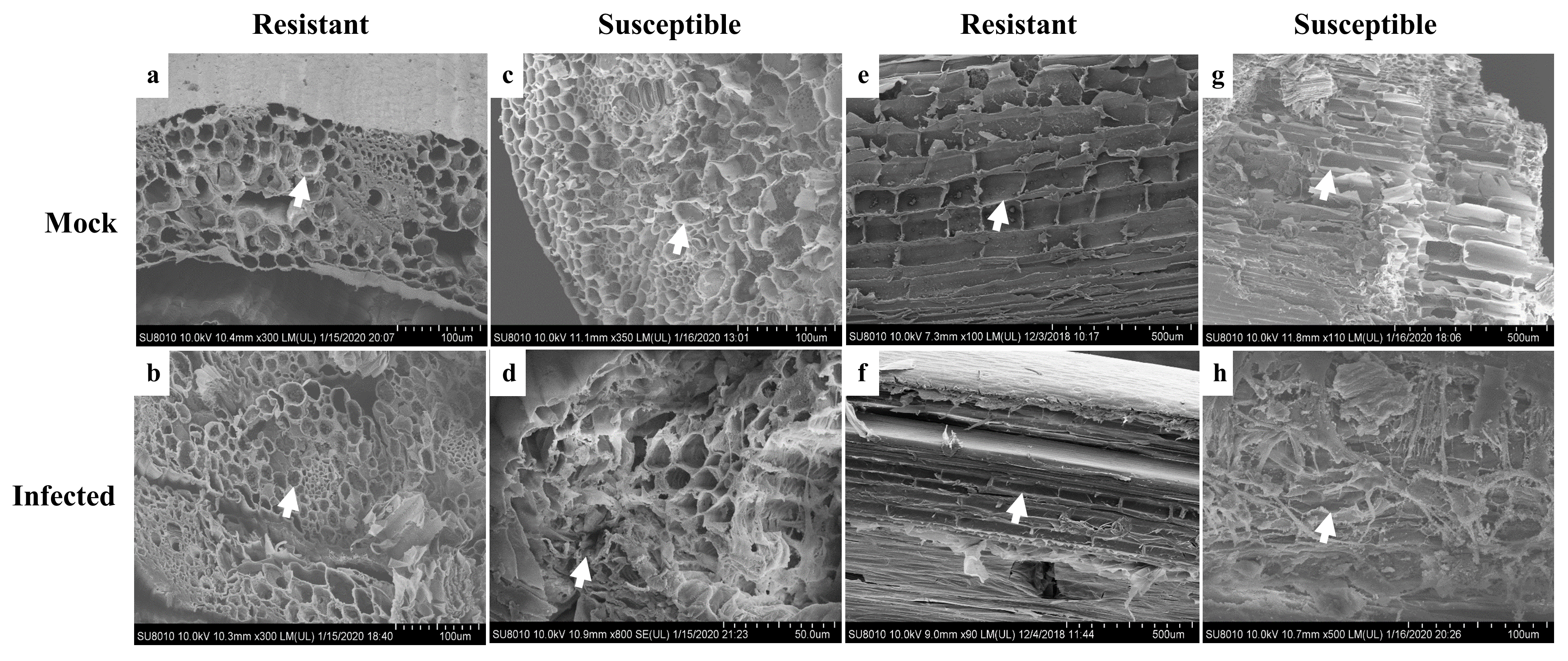

Supplement: Supplementary file 8 — Additional file 8: Fig. S8. Histological characteristics of the stems of the mock and infected resistant and susceptible cultivars at the tillering stage (Z21) under scanning electron microscopy. (a) Stem cell structure of the mock resistant cultivar. (b) Stem cell structure of the infected resistant cultivar. (c) Stem cell structure of the mock susceptible cultivar. (d) Stem cell structure of the infected susceptible cultivar. (e) Longitudinal section of the stem of the mock resistant cultivar. (f) Longitudinal section of the stem of the infected resistant cultivar. (g) Longitudinal section of the stem of the mock susceptible cultivar. (h) Longitudinal section of the stem of the infected susceptible cultivar. The resistant cultivar was Yinong 18/Lankao 8, and the susceptible cultivar was Dongxuan 3. White arrows in (a)(b)(c)(d) indicate stem cells and white arrows in (e)(f)(g)(h) indicate longitudinal section stem cells. [file 12870_2020_2819_MOESM8_ESM.tif]

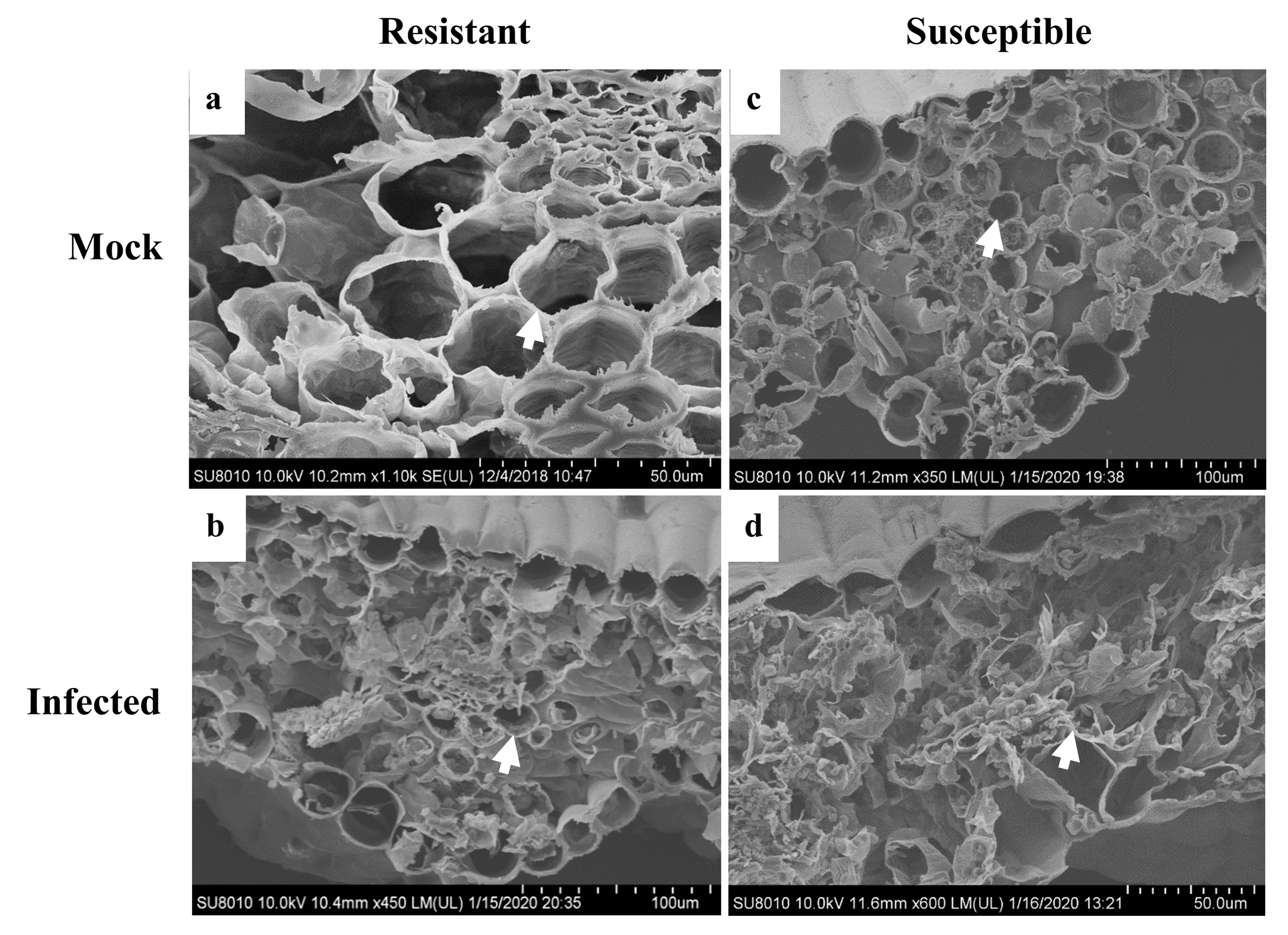

Supplement: Supplementary file 9 — Additional file 9: Fig. S9. Histological characteristics of the leaves of the mock and infected resistant and susceptible cultivars at the tillering stage (Z21) under scanning electron microscopy. (a) Mesophyll cells of the mock resistant cultivar. (b) Mesophyll cells of the infected resistant cultivar. (c) Mesophyll cells of the mock susceptible cultivar. (d) Mesophyll cells of the infected susceptible cultivar. The resistant cultivar was Yinong 18/Lankao 8, and the susceptible cultivar was Dongxuan 3. White arrows in (a)(b)(c)(d) indicate mesophyll cells. [file 12870_2020_2819_MOESM9_ESM.tif]

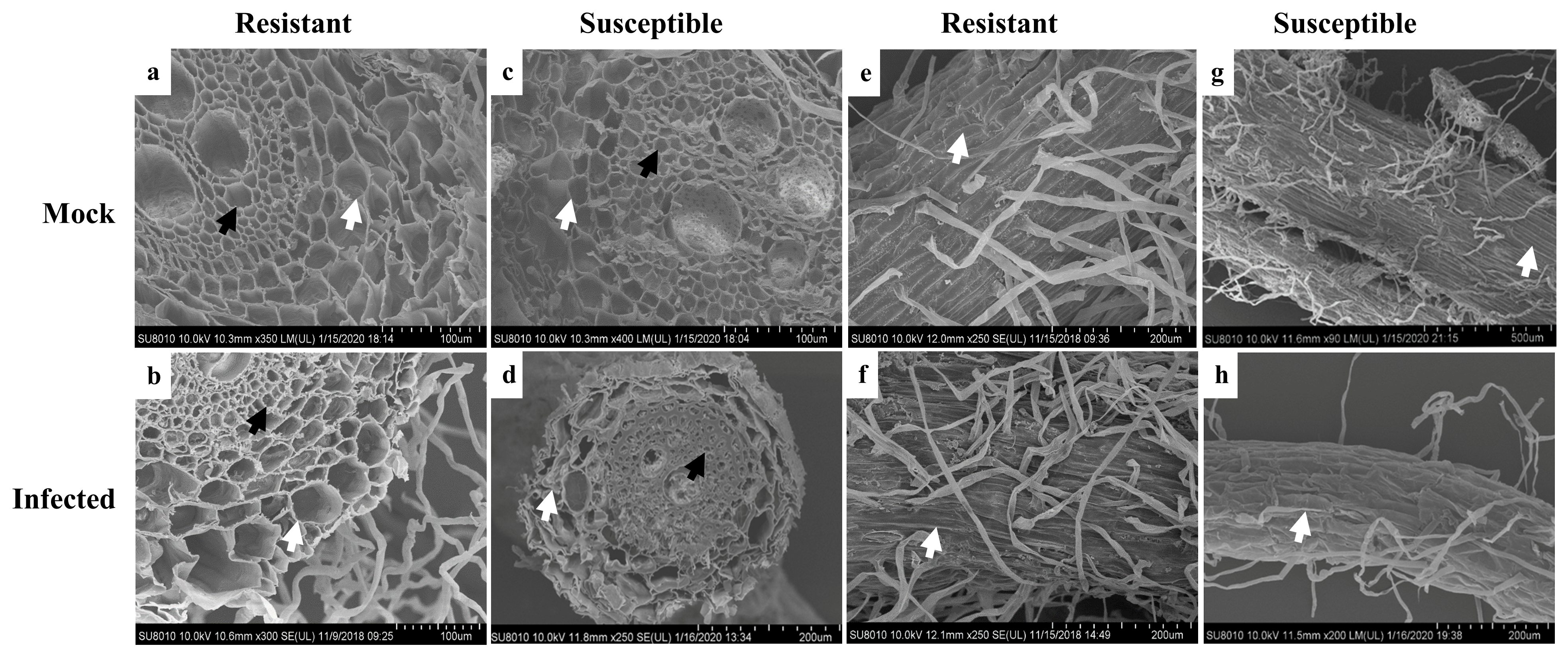

Supplement: Supplementary file 10 — Additional file 10: Fig. S10. Histological characteristics of the roots of the mock and infected resistant and susceptible cultivars at the seedling growth stage (Z13) under scanning electron microscopy. (a) Vascular bundle cells and cortical parenchyma cells of the mock resistant cultivar. (b) Vascular bundle cells and cortical parenchyma cells of the infected resistant cultivar. (c) Vascular bundle cells and cortical parenchyma cells of the mock susceptible cultivar. (d) Vascular bundle cells and cortical parenchyma cells of the infected susceptible cultivar. (e) Root epidermal cells of the mock resistant cultivar. (f) Root epidermal cells of the infected resistant cultivar. (g) Root epidermal cells of the mock susceptible cultivar. (h) Root epidermal cells of the infected susceptible cultivar. The resistant cultivar was Yinong 18/Lankao 8, and the susceptible cultivar was Dongxuan 3. White arrows in (a)(b)(c)(d) indicate cortical parenchyma cells, black arrows in (a)(b)(c)(d) indicate vascular bundle cells and white arrows in (e)(f)(g)(h) indicate root epidermal cells. [file 12870_2020_2819_MOESM10_ESM.tif]

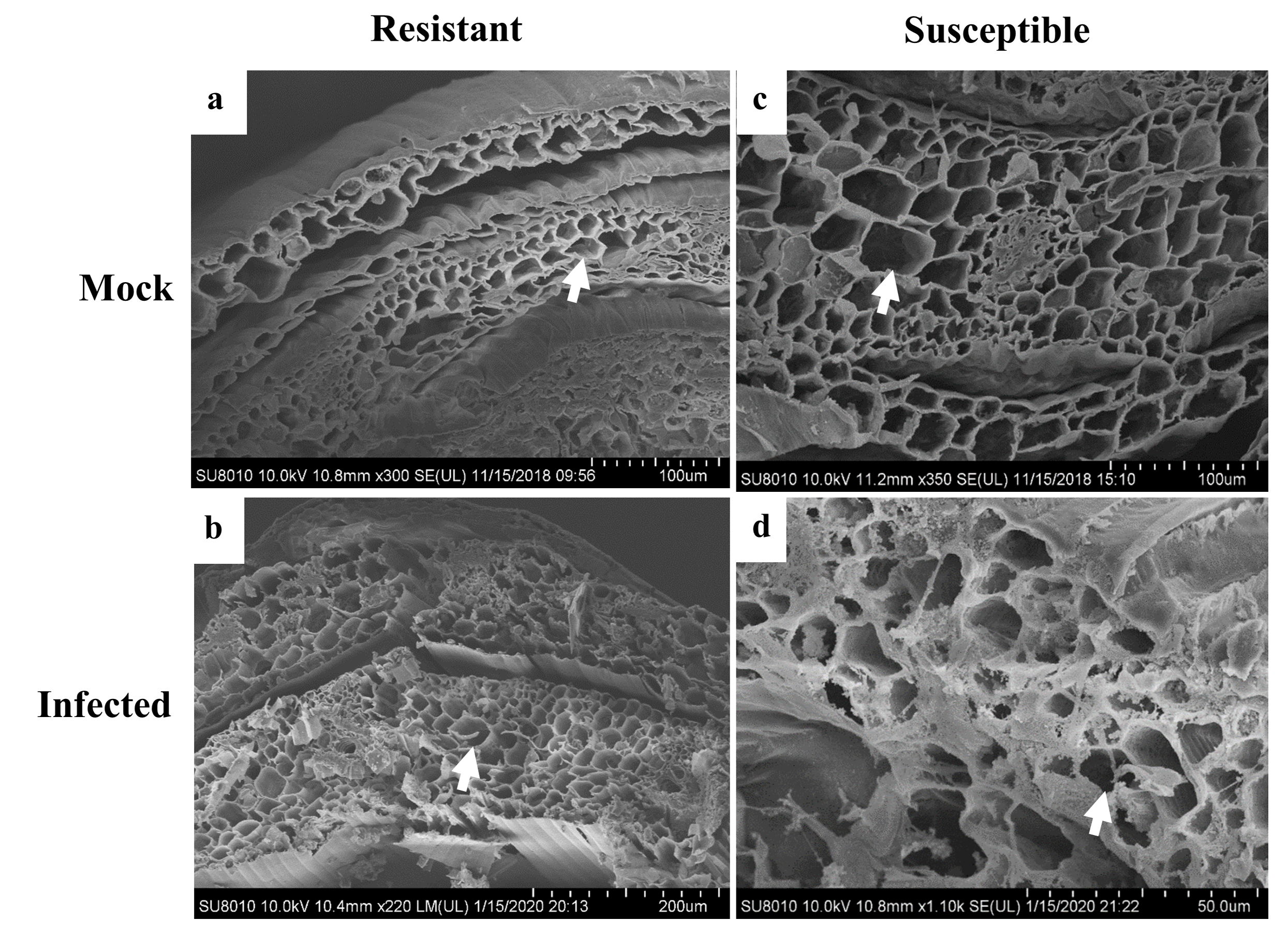

Supplement: Supplementary file 11 — Additional file 11: Fig. S11. Histological characteristics of the stems of the mock and infected resistant and susceptible cultivars at the seedling growth stage (Z13) under scanning electron microscopy. (a) Stem cell structure of the mock resistant cultivar. (b) Stem cell structure of the infected resistant cultivar. (c) Stem cell structure of the mock susceptible cultivar. (d) Stem cell structure of the infected susceptible cultivar. The resistant cultivar was Yinong 18/Lankao 8, and the susceptible cultivar was Dongxuan 3. White arrows in (a)(b)(c)(d) indicate stem cells. [file 12870_2020_2819_MOESM11_ESM.tif]

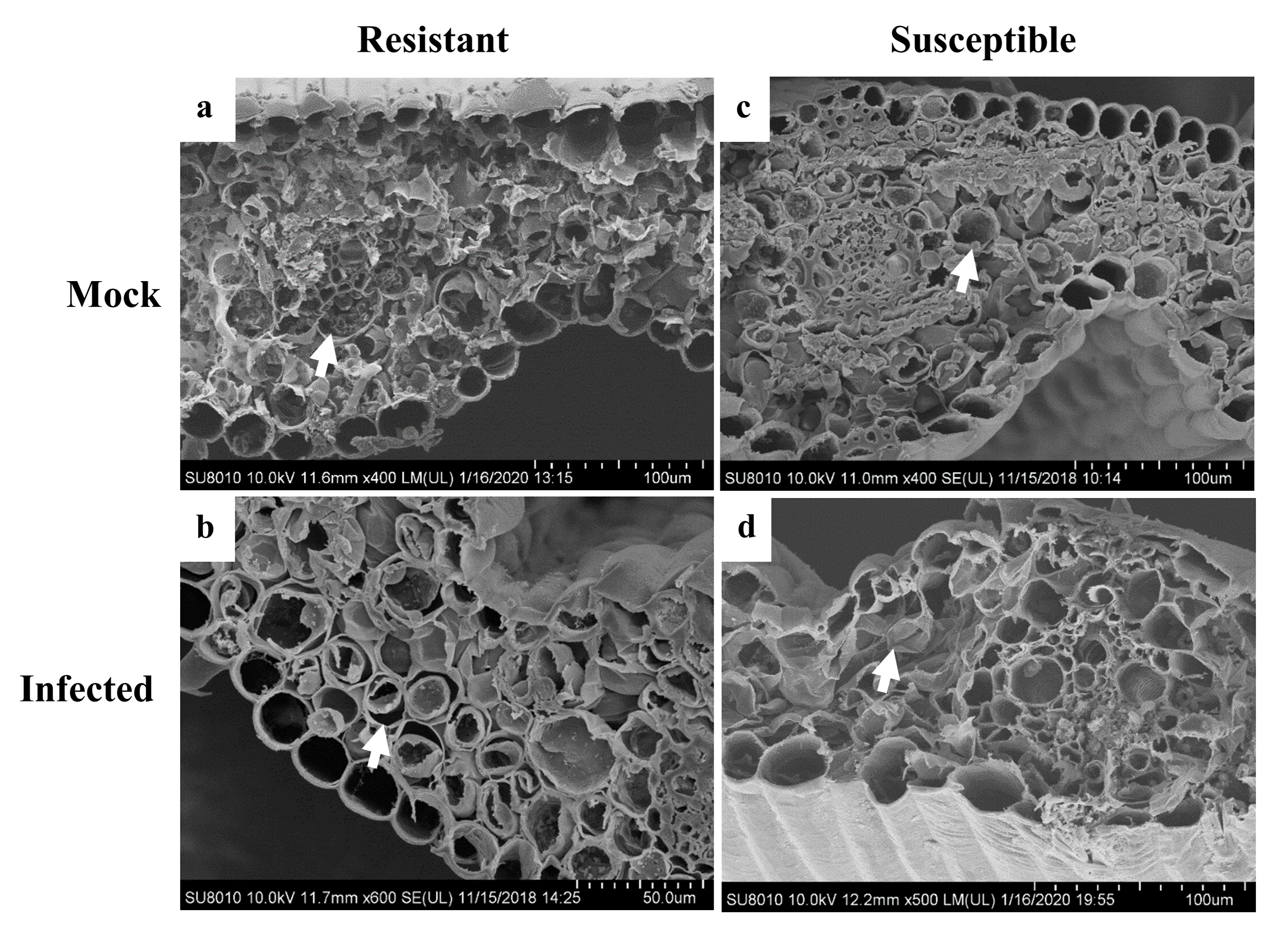

Supplement: Supplementary file 12 — Additional file 12: Fig. S12. Histological characteristics of the leaves of the mock and infected resistant and susceptible cultivars at the seedling growth stage (Z13) under scanning electron microscopy. (a) Mesophyll cells of the mock resistant cultivar. (b) Mesophyll cells of the infected resistant cultivar. (c) Mesophyll cells of the mock susceptible cultivar. (d) Mesophyll cells of the infected susceptible cultivar. The resistant cultivar was Yinong 18/Lankao 8, and the susceptible cultivar was Dongxuan 3. White arrows in (a)(b)(c)(d) indicate mesophyll cells. [file 12870_2020_2819_MOESM12_ESM.tif]

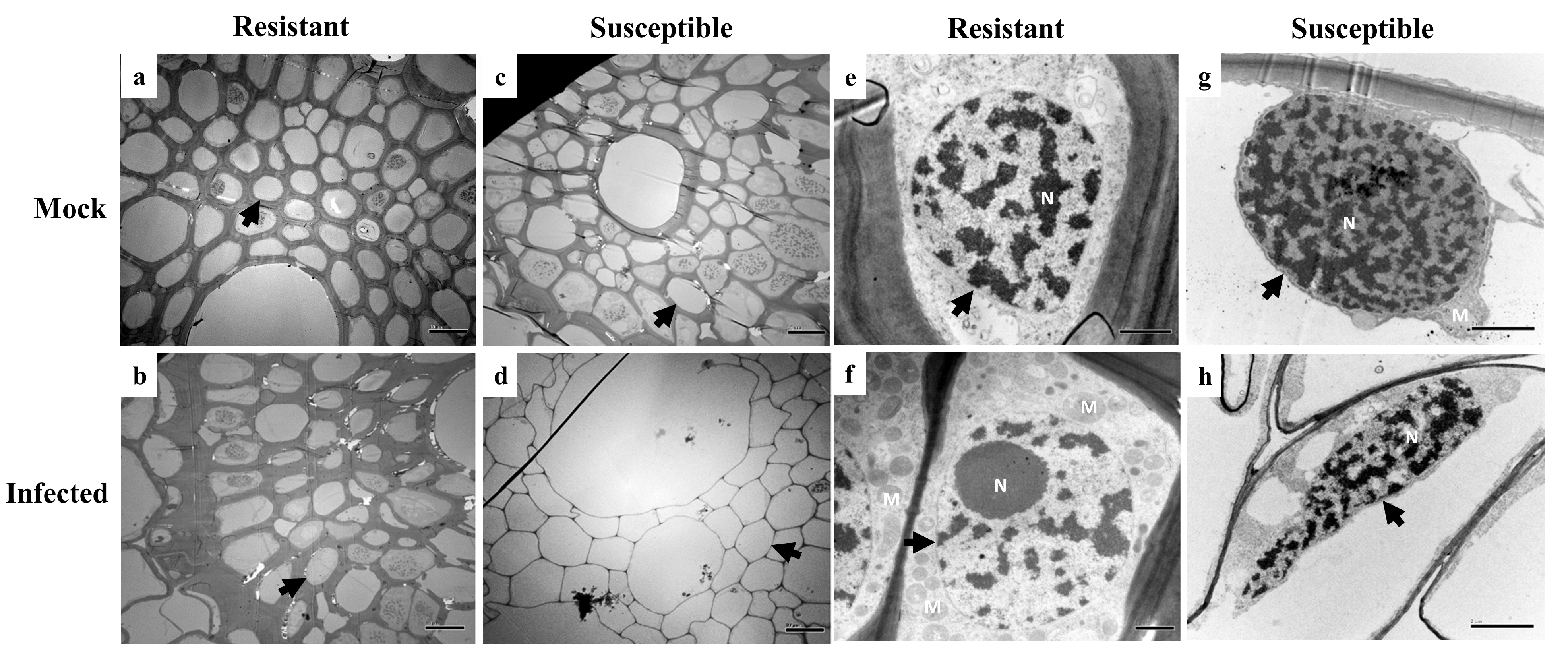

Supplement: Supplementary file 13 — Additional file 13: Fig. 13. Histological characteristics of the roots of the mock and infected resistant and susceptible cultivars at the seedling growth stage (Z13) under transmission electron microscopy. (a) Vascular bundle cells of the mock resistant cultivar. (b) Vascular bundle cells of the infected resistant cultivar. (c) Vascular bundle cells of the mock susceptible cultivar. (d) Vascular bundle cells of the infected susceptible cultivar. (e) Root nucleus of the mock resistant cultivar. (f) Root nucleus of the infected resistant cultivar. (g) Root nucleus of the mock susceptible cultivar. (h) Root nucleus of the infected susceptible cultivar. The resistant cultivar was Mianyang 26/Yumai 47, and the susceptible cultivar was CU42. Black arrows in (a)(b)(c)(d) indicate vascular bundle cells, and black arrows in (e)(f)(g)(h) indicate root nucleus. (N: Nucleus; M: Mitochondrion; scale bar of (a)(b)(c)(d) =10 μm; scale bar of (e)(f) =1 μm; scale bar of (g)(h) =2 μm). [file 12870_2020_2819_MOESM13_ESM.tif]

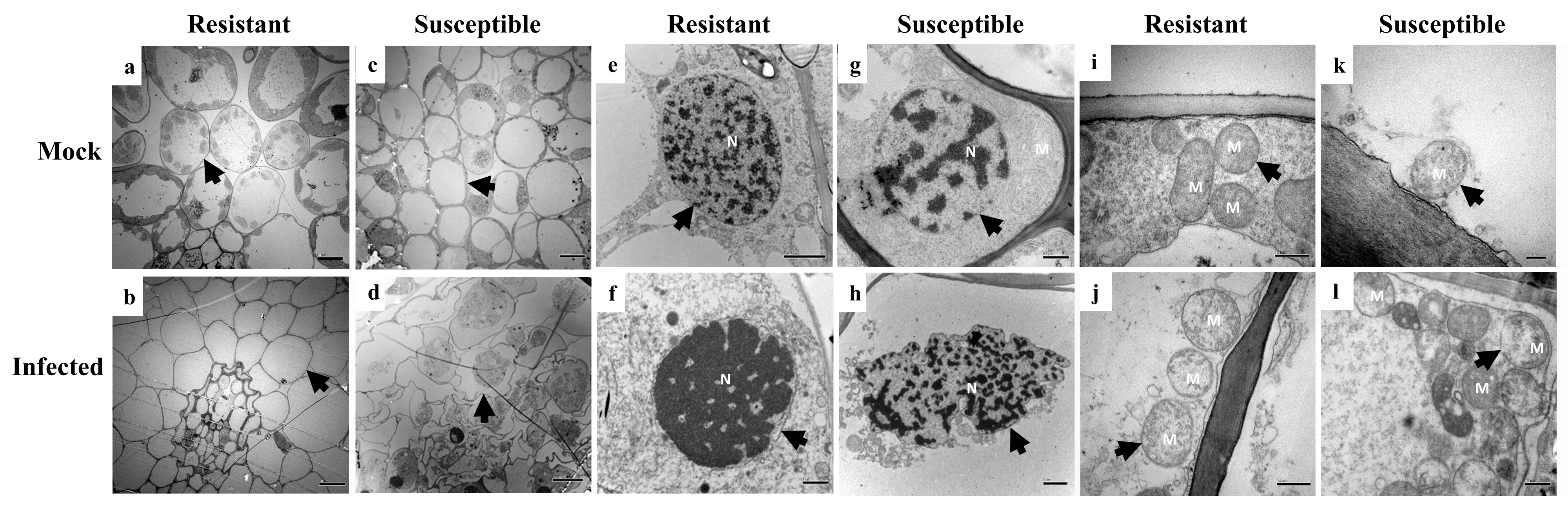

Supplement: Supplementary file 14 — Additional file 14: Fig. S14. Histological characteristics of stem cells of the mock and infected resistant and susceptible cultivars at the seedling growth stage (Z13) under transmission electron microscopy. (a) Stem cell structure of the mock resistant cultivar. (b) Stem cell structure of the infected resistant cultivar. (c) Stem cell structure of the mock susceptible cultivar. (d) Stem cell structure of the infected susceptible cultivar. (e) Stem cell nucleus of the mock resistant cultivar. (f) Stem cell nucleus of the infected resistant cultivar. (g) Stem cell nucleus of the mock susceptible cultivar. (h) Stem cell nucleus of the infected susceptible cultivar. (i) Stem mitochondrion of the mock resistant cultivar. (j) Stem mitochondrion of the infected resistant cultivar. (k) Stem mitochondria of the mock susceptible cultivar. (l) Stem mitochondrion of the infected susceptible cultivar. The resistant cultivar was Mianyang 26/Yumai 47, and the susceptible cultivar was CU42. Black arrows in (a)(b)(c)(d) indicate stem cells, black arrows in (e)(f)(g)(h) indicate stem cell nucleus, and black arrows in (i)(j)(k)(l) indicate stem mitochondrion. (N: Nucleus; M: Mitochondrion; scale bar of (a)(b)(c)(d) =10 μm; scale bar of (e)(f)(g)(h) =1 μm; scale bar of (i)(j)(k)(l) =0.5 μm). [file 12870_2020_2819_MOESM14_ESM.tif]

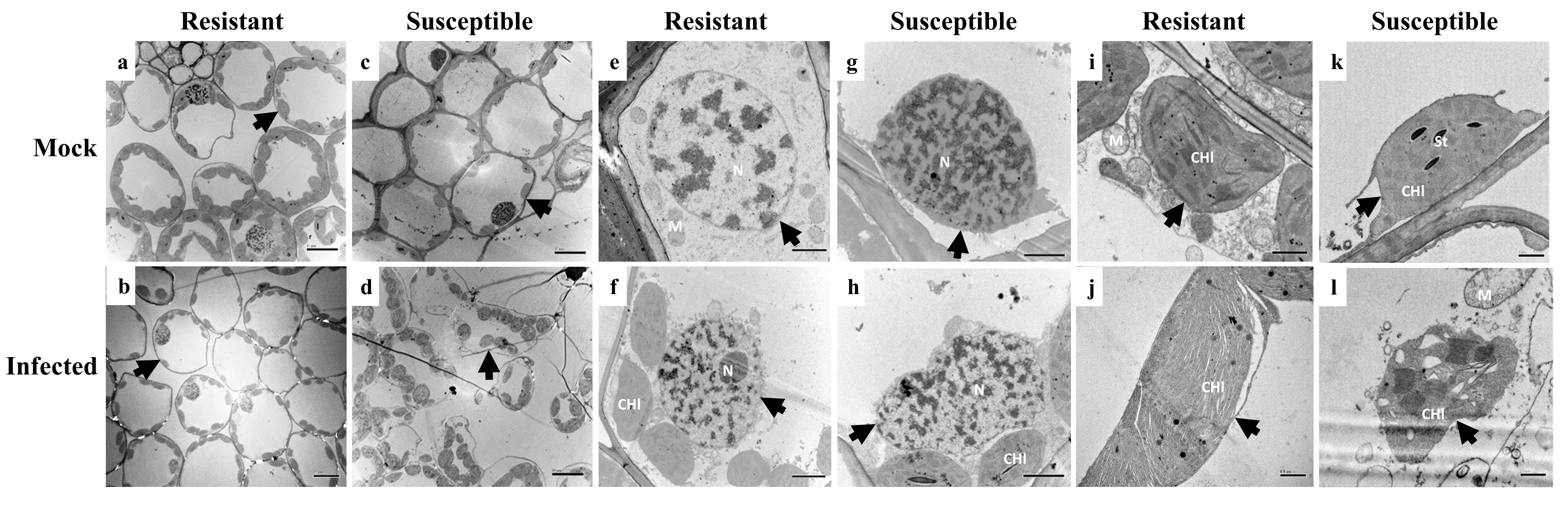

Supplement: Supplementary file 15 — Additional file 15: Fig. S15. Histological characteristics of the leaves of the mock and infected resistant and susceptible cultivars at the seedling growth stage (Z13) under transmission electron microscopy. (a) Mesophyll cell structure of mock resistant plants. (b) Mesophyll cell structure of infected resistant plants. (c) Mesophyll cell structure of mock susceptible plants. (d) Mesophyll cell structure of infected susceptible plants. (e) Nucleus of mock resistant plants. (f) Nucleus of infected resistant plants. (g) Nucleus of mock susceptible plants. (h) Nucleus of infected susceptible plants. (i) Chloroplast of mock resistant plants. (j) Chloroplast of infected resistant plants. (k) Chloroplast of mock susceptible plants. (l) Chloroplast of infected susceptible plants. The resistant cultivar was Mianyang 26/Yumai 47 and the susceptible cultivar was CU42. Black arrows in (a)(b)(c)(d) indicate mesophyll cells, black arrows in (e)(f)(g)(h) indicate leaf nucleus, black arrows in (a)(b)(c)(d) indicate stem cells, and black arrows in (i)(j)(k)(l) indicate leaf chloroplasts (N: Nucleus; CHl: Chloroplast; St: Starch granule; M: Mitochondrion; scale bar of (a)(b)(c)(d) =10 μm; scale bar of (e)(f)(g)(h) =1 μm; scale bar of (i)(j)(k)(l) =0.5 μm). [file 12870_2020_2819_MOESM15_ESM.tif]

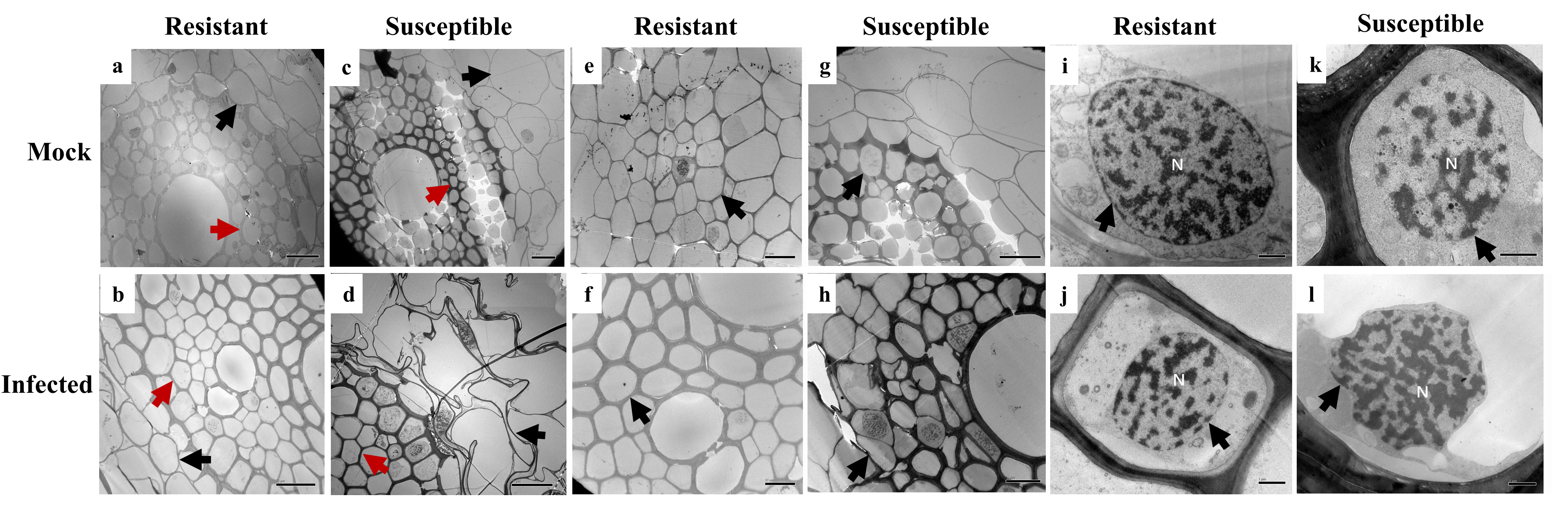

Supplement: Supplementary file 16 — Additional file 16: Fig. S16. Histological characteristics of the roots of the mock and infected resistant and susceptible cultivars at the tillering stage (Z21) under transmission electron microscopy. (a) Vascular bundle cells and cortical parenchyma cells of the mock resistant cultivar. (b) Vascular bundle cells and cortical parenchyma cells of the infected resistant cultivar. (c) Vascular bundle cells and cortical parenchyma cells of the mock susceptible cultivar. (d) Vascular bundle cells and cortical parenchyma cells of the infected susceptible cultivar. (e) Root cell contents of the mock resistant cultivar. (f) Root cell contents of the infected resistant cultivar. (g) Root cell contents of the mock susceptible cultivar. (h) Root cell contents of the infected susceptible cultivar. (i) Root nucleus of the mock resistant cultivar. (j) Root nucleus of the infected resistant cultivar. (k) Root nucleus of the mock susceptible cultivar. (l) Root nucleus of the infected susceptible cultivar. The resistant cultivar was Yinong 18/Lankao8 and the susceptible cultivar was Dongxuan 3. Black arrows in (a)(b)(c)(d) indicate cortical parenchyma cells, red arrows in (a)(b)(c)(d) indicate vascular bundle cells, black arrows in (e)(f)(g)(h) indicate root cell contents, and black arrows in (i)(j)(k)(l) indicate root nuclei (N: Nucleus; M: Mitochondrion; scale bar of (a)(b)(c)(d)(g) = 20 μm; scale bar of (e)(f) (h) =10 μm; scale bar of (i)(j) (k)(l) =1 μm). [file 12870_2020_2819_MOESM16_ESM.tif]
